# A Compendium of Preparation and Application of Stem Cells in Parkinson's Disease: Current Status and Future Prospects

**DOI:** 10.3389/fnagi.2016.00117

**Published:** 2016-05-31

**Authors:** Yan Shen, Jinsha Huang, Ling Liu, Xiaoyun Xu, Chao Han, Guoxin Zhang, Haiyang Jiang, Jie Li, Zhicheng Lin, Nian Xiong, Tao Wang

**Affiliations:** ^1^Department of Neurology, Tongji Medical College, Union Hospital, Huazhong University of Science and TechnologyWuhan, China; ^2^Department of Psychiatry, Harvard Medical School, Division of Alcohol and Drug Abuse, and Mailman Neuroscience Research Center, McLean HospitalBelmont, MA, USA

**Keywords:** Parkinson's Disease, dopaminergic (DA) neurons, stem cells, transplantation, preparation, cell replacement therapy (CRT)

## Abstract

Parkinson's Disease (PD) is a progressively neurodegenerative disorder, implicitly characterized by a stepwise loss of dopaminergic (DA) neurons in the substantia nigra pars compacta (SNpc) and explicitly marked by bradykinesia, rigidity, resting tremor and postural instability. Currently, therapeutic approaches available are mainly palliative strategies, including L-3,4-dihydroxy-phenylalanine (L-DOPA) replacement therapy, DA receptor agonist and deep brain stimulation (DBS) procedures. As the disease proceeds, however, the pharmacotherapeutic efficacy is inevitably worn off, worse still, implicated by side effects of motor response oscillations as well as L-DOPA induced dyskinesia (LID). Therefore, the frustrating status above has propeled the shift to cell replacement therapy (CRT), a promising restorative therapy intending to secure a long-lasting relief of patients' symptoms. By far, stem cell lines of multifarious origins have been established, which can be further categorized into embryonic stem cells (ESCs), neural stem cells (NSCs), induced neural stem cells (iNSCs), mesenchymal stem cells (MSCs), and induced pluripotent stem cells (iPSCs). In this review, we intend to present a compendium of preparation and application of multifarious stem cells, especially in relation to PD research and therapy. In addition, the current status, potential challenges and future prospects for practical CRT in PD patients will be elaborated as well.

## Introduction

Parkinson's Disease (PD) is one of the most prevalent neurodegenerative disorders, second only to Alzheimer's Disease, affecting approximately 1% of the population over the age of 60 and 4% over 80 (de Lau and Breteler, 2006). It is a progressively deteriorative disorder characterized by the stepwise loss of dopaminergic (DA) neurons in the substantia nigra pars compacta (SNpc), a region in the ventral midbrain (VM), and neurons from other regions of the peripheral and central nervous systems (CNS). During the unbeknown preclinical course, degeneration of non-DA neurons in the brainstem, olfactory bulb and cortex has already occurred insidiously. Moreover, the peripheral nervous systems, once involved, incur similar pathological changes as well, for instance, in the gut and heart (Braak et al., 2003; Sundberg and Isacson, 2014). Because of the importance of nigrostriatal DA neurons in dominating somatic movement, PD is chiefly marked by bradykinesia, rigidity, resting tremor, and postural instability. However, other non-motor manifestations such as anxiety, passivity, depression, psychosis, dementia, and sleep disturbance also set out to emerge insidiously as the disease progresses (Chaudhuri et al., 2006). As a result of the systemic involvement and multifunctional impairment, therefore, PD has been described as a clinical syndrome (Fahn, 2003). Currently, therapeutic approaches available are mainly aiming to relieve PD motor symptoms including L-3,4-dihydroxy-phenylalanine (L-DOPA) replacement therapy, administration of DA agonist, and deep brain stimulation (DBS; Foltynie and Hariz, 2010) in subthalamic nucleus and globus pallidus via surgically implanted electrodes (Politis and Lindvall, 2012), all of which are palliative and incapable of counteracting the progression course. Moreover, as the disease proceeds, the efficacy of pharmacotherapy is gradually undermined, implicated by the development of various types of motor response oscillations such as on-off, wearing off phenomena, as well as L-DOPA induced dyskinesia (LID; Poewe et al., 2010). Up to now, there still lack restorative treatments for PD, which plus the frustrating therapeutic status partly account for why current research efforts conformably shift to the field of stem cell research.

**Figure 1:**
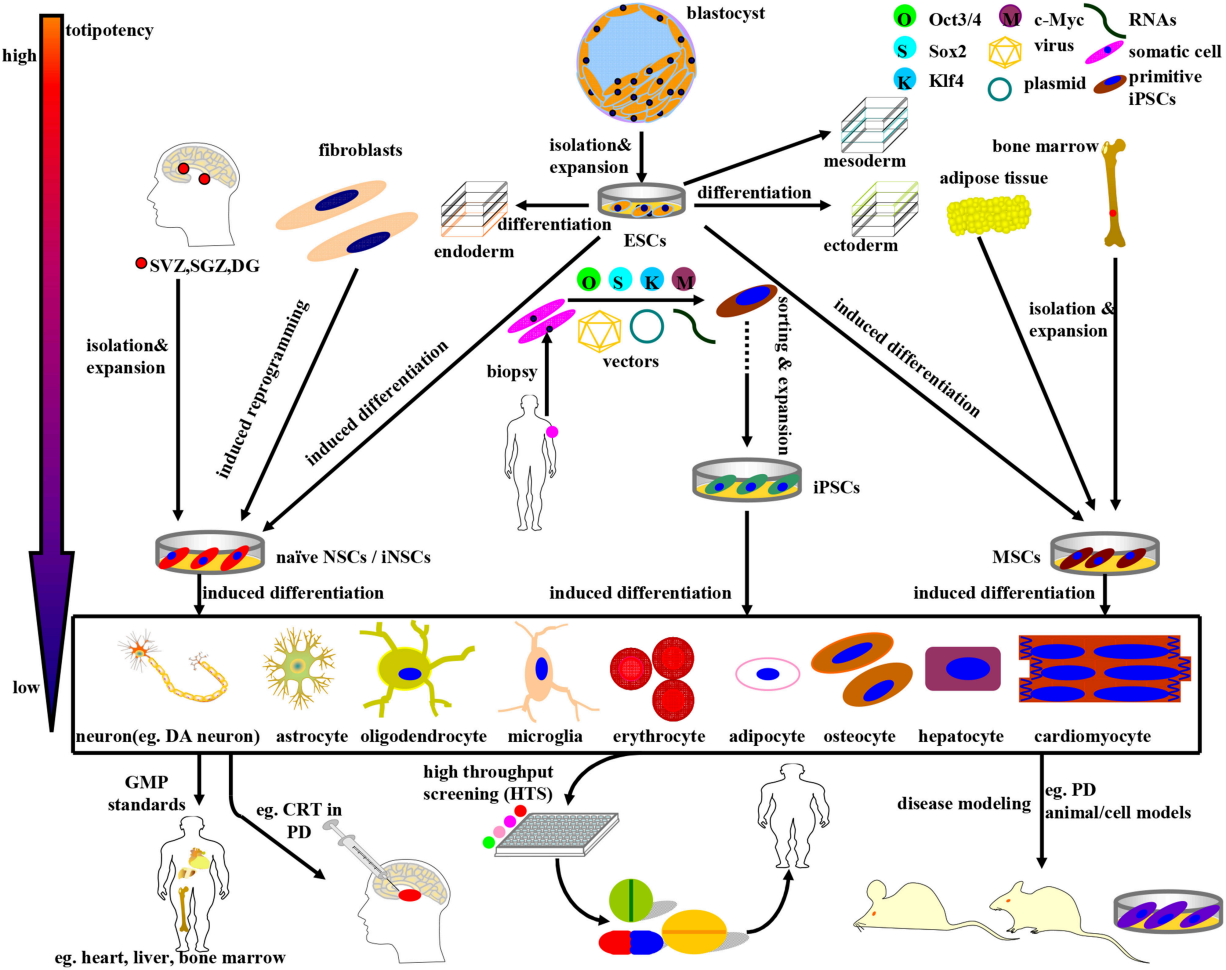
**Schematic illustration of the derivation, differentiation, and application of stem cells currently available in PD research and therapy**. The stem cells above can be divided into four categories: ESCs, NSCs, MSCs, and iPSCs, accompanying a gradually declining totipotency. (1) ESCs, mainly derived from blastocyst inner mass, can differentiate into endoderm, mesoderm, and ectoderm simultaneously under normal circumstances. In certain cases, ESCs can be induced to differentiate into NSCs and MSCs as well. (2) NSCs, isolated directly from specific brain niches or reprogrammed from fibroblasts, can fulfill neural lineage differentiation into neurons and almost all neuroglia cells. (3) MSCs, primarily derived from mesenchymal tissues, can commitedly differentiate into almost all cells of mesodermal origins. Noteworthy, MSCs can be induced to differentiate into DA neurons as well under specific combinations of induction protocols. (4) iPSCs, a promising stem cell source with multi-lineage differentiation potency, can be reprogrammed from adult human somatic cells (such as fibroblasts) by retro-virally introducing the classical OSKM (Oct3/4, Sox2, Klf4, and c-Myc) transcription factors. Guided by the GMP standards, the stem cells above and the terminally differentiated cells can be further sorted, purified, and expanded so as to be applied to disease modeling, drug screening, and CRT practice. For example, ESCs, MSCs, NSCs, and DA neurons can be employed in (i) PD models preparation; (ii) potential drugs screening; (iii) CRT treatment of PD.

By definition, cells that can self-renew and produce progenies as well as differentiating into multiple cell lineages are termed as stem cells (Lunn et al., 2011). Since proposal of the concept, subsequently yielded research achievement has enabled the establishment of multifarious stem cell lines, many of which have been employed for DA neurons derivation and differentiation so as to be applied to disease modeling, drug screening, and cell replacement (sometimes termed as “cell transplantation”) therapy (CRT) for PD (see Figure 1). At present, the most commonly studied stem cell sources for DA neuron derivation are human fetal ventral midbrain (hfVM) cells, human embryonic stem cells (hESCs), human neural stem/precursor/ progenitor cells (hNSCs/hNPCs), human mesenchymal stem cells (hMSCs), human induced neural stem cells (hiNSCs), and human-induced pluripotent stem cells (hiPSCs; Pittenger et al., 1999; Cooper et al., 2010; Hargus et al., 2010; Kriks et al., 2011; Kirkeby et al., 2012; Sundberg et al., 2013), which hold great promise to be restorative therapeutic regimens (see Figure 1). In relation to PD, an estimation of 30% DA cells loss in the SN combined with a greater than 60% reduction of DA neurons innervation to the striatum can result in the emergence of clinical features (Cheng et al., 2010). Thus, any type of CRT protocol needs to restore the nigrostriatal system at least back to 30% of normal level so as to be effective. To warrant future standardization and homogenization of stem cell-based CRT, the ideal appraisal criteria should conform to the protocols below (Laguna Goya et al., 2008):

1. Yielding sufficient neural lineage specific cell lines available for transplantation;
2. Sustained survival (i.e., years) of the cells post implantation;
3. Differentiation into nigral DA neurons in sufficient numbers (approximately 100,000) with evidence for release of dopamine from the grafted cells;
4. Behavioral recovery consequent upon the transplantation of these cells;
5. No evidence of tumor formation or differentiation into neurons/cells that disrupt the nigrostriatal circuits further;
6. Subordinate to Good Manufacturing Practice (GMP) standards;

Here in this review, we propose to present an elaborate compendium of the preparation and application of stem cell lines, especially ESCs, NSCs/NPCs, iNSCs, MSCs, and iPSCs for research and therapy of PD (see Figure 1). As a result of the more superior pluripotency, more distinctive differentiation features and more approbatory sources, iPSCs appear to be a more preferential CRT candidate. In addition, the current status, future prospects and CRT related inflammation issues for practical stem cell based therapy in PD patients will be elaborated as well.

## Embryonic stem cells (ESCs)

ESCs, derived from the inner mass of a developing blastocyst, have the potential to self-renew and give rise into cells of all three primary germ layers-ectoderm, mesoderm and endoderm under certain normal circumstances (see Figure 1). Inspired by the fascinating differentiation potencies, murine and human ESCs have been established in succession (Evans and Kaufman, 1981; Martin, 1981; Thomson et al., 1998). In the context of the initial derivation of human ESCs (hESCs), essential characteristics of primate ESCs have been proposed to include the features below: (i) derivation from the pre-implantation or peri-implantation embryo; (ii) prolonged undifferentiated proliferation; and (iii) stable developmental potential to form derivatives of all three embryonic germ layers even after prolonged culture (Thomson et al., 1998). Given that hESCs have the ability to provide a virtually limitless supply of homogenous DA progenitors/precursor cells and neurons of specific neural lineages, neural transplantation of this cell lines is a promising strategy to restore DA dysfunction and modify disease progression in PD.

**Figure 2:**
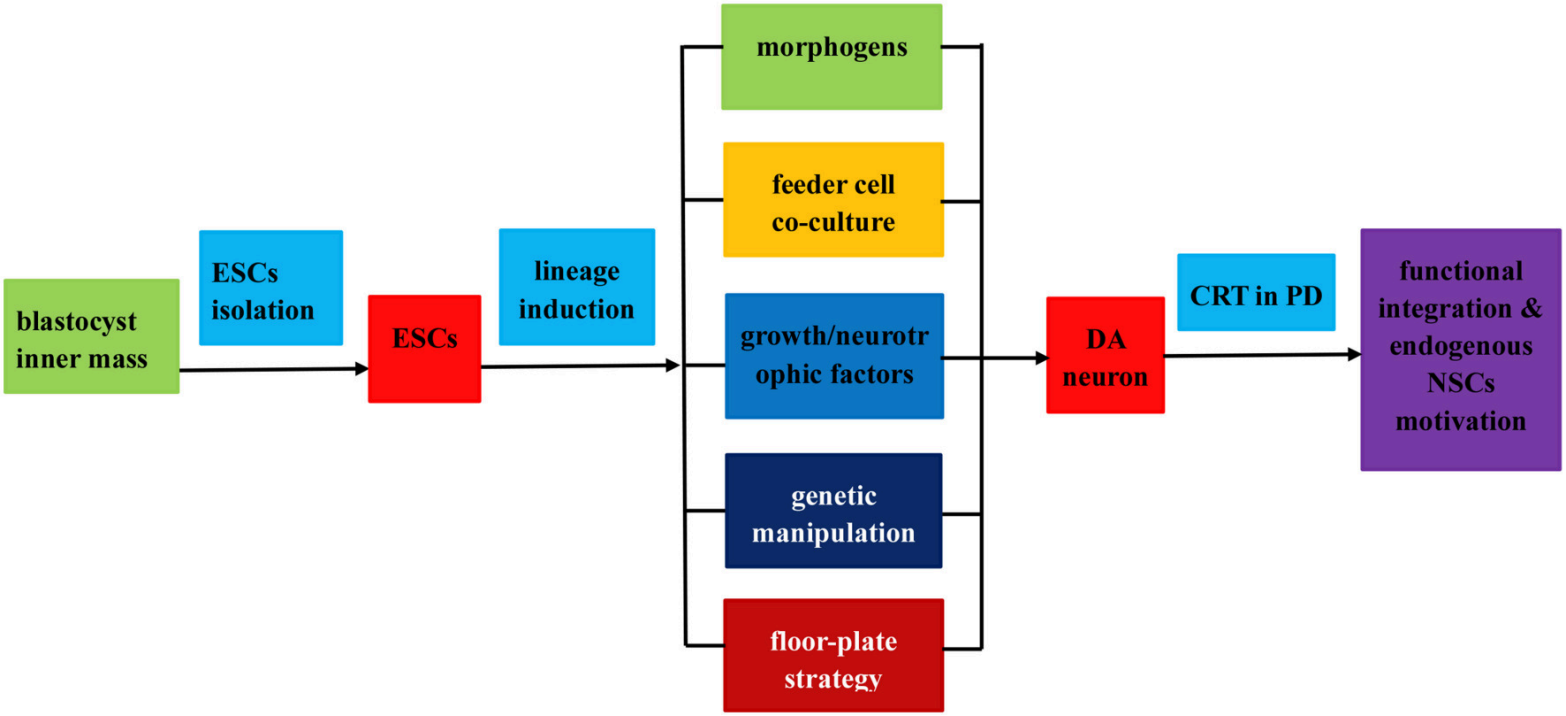
**Flow chart of the isolation, induced differentiation, and application as a renewable replacement cell source in PD treatment**. The entire procedures fall into three steps as follows: (1) ESCs isolation from blastocyst inner mass; (2) induced differentiation of ESCs into DA neurons by virtue of (i) application of morphogens or soluble growth factors; (ii) feeder cell co-culture; (iii) genetic manipulation; (iv) floor plate lineage induction strategy; (3) transplantation of the induced DA neurons in PD treatment via functional integration and endogenous NPCs motivation.

Attempts to induce further differentiation of ESCs *in vitro* involve several different approaches (see Figure 2). For instance, a significant improvement of neural lineages induction achieved by application of several morphogens such as all-trans retinoic acid (RA), sonic hedgehog (SHH), fibroblast growth factor (FGF), epidermal growth factor (EGF), bone morphogentic proteins (BMPs), and glial cell derived neurotrophic factor (GDNF; Fraichard et al., 1995; Ciccolini and Svendsen, 1998; Guan et al., 2001; Buytaert-Hoefen et al., 2004; Perrier et al., 2004; Li et al., 2005), all employed as neurogenic stimulators which are essential for normal embryonic development and differentiation as well (Ross et al., 2000; see Table 1). Apart from morphogens above, there exist several tissue culture protocols available to induce production of A9 DA neurons from hESCs, including co-culturing feeder cells (Kawasaki et al., 2000; Perrier et al., 2004; Zeng et al., 2004; Park et al., 2005; Brederlau et al., 2006), soluble growth factors (Lee et al., 2000; Schulz et al., 2004; Takagi et al., 2005; Yan et al., 2005; Yang et al., 2008), genetic manipulation (Kim et al., 2002; Chung et al., 2005; Andersson et al., 2006) and specific combination of the methods above (see Table 1). One method involves co-culturing ESCs with feeder cells that possess stromal cell derived inducing activity (SDIA). Co-culturing mouse PA6 stromal cells with murine and human ESCs have been demonstrated to induce differentiation of DA neurons, nevertheless, with different percentage of TH+ (tyrosine hydroxylase, a critical enzyme involved in DA synthesis) neurons (Kawasaki et al., 2000; Zeng et al., 2004; Brederlau et al., 2006). Besides, a number of soluble growth factors and chemicals such as ascorbic acid, cAMP, TGF-beta3, BDNF (brain-derived neurotrophic factor) and GDNF are also capable of inducing differentiation of ESCs into beta-tubulin III+/TH+ DA neurons (Lee et al., 2000; Schulz et al., 2004; Takagi et al., 2005; Yan et al., 2005; Yang et al., 2008). Moreover, transplantation of the induced DA neurons into PD animal models can relieve its functional deficits (Schulz et al., 2004; Takagi et al., 2005; Yan et al., 2005; Yang et al., 2008). Of note, combining soluble growth factors with feeder cell have efficiently produced an enriched population of midbrain DA neurons as well (Perrier et al., 2004; Park et al., 2005; Roy et al., 2006; Sonntag et al., 2007). In addition, it is feasible to successfully facilitate the differentiation of ESCs to certain lineages by genetic manipulation consisting of specific activation of key fate-determining transcription factors such as Nurr1, Lmx1a, Pitx3, Pax4, and GATA (Zetterstrom et al., 1997; Castillo et al., 1998; Saucedo-Cardenas et al., 1998; Fujikura et al., 2002; Kim et al., 2002; Blyszczuk et al., 2003; Chung et al., 2005; Andersson et al., 2006), among which Nurr1, Lmx1a, and Pitx3 can facilitate the induction of midbrain DA neurons from murine ESCs (mESCs; Kim et al., 2002; Chung et al., 2005; Andersson et al., 2006). Better yet, a rapid and concise protocol employing wholly chemically defined human additives such as SHH, FGF8 (Yan et al., 2005) or recombinant human noggin, bFGF (basic FGF), dibutyryl–cAMP (Iacovitti et al., 2007) or FGF8b and SHH (Yang et al., 2008), omitting the collaboration of feeder cells and transcription factors, have successfully facilitated the differentiation of hESCs into DA neurons. As a matter of fact, development of midbrain DA neurons is tightly orchestrated by a cluster of transcription factors (such as OTX2, LMX1a, FOXa2, LMX1b, MSX1, EN1, NGN2, NURR1, and PITX3) and signaling molecules (such as SHH, WNT, and FGF8) apart from synergism of SDIA labeled feeder cell, specifically instructing the differentiation into DA neurons. Moreover, a recent elegant floor-plate based strategy involving tight temporal control of key factor exposure of cultures have yielded TH+ neurons exhibiting A9 phenotype with higher reprogramming efficiency and shorter period (Kriks et al., 2011; Ryan et al., 2013). Therefore, joint employment of stromal feeder cell, genetic manipulation signaling molecules and elaborate regulation of key factor exposure in culture system can synergistically facilitate the induction of DA neurons from ESCs. As for the CRT of ESCs in PD, apart from *in vivo* functional integration, transplanted cells may stimulate neurogenesis through recruitment and activation of endogenous NPCs (Tomaskovic-Crook and Crook, 2011; see Figure 2).

**Table 1:** **Existing DA neurons differentiation protocols and corresponding application in PD models of ESCs, NSCs/NPCs, and MSCs**.

**Stem cell sources**	**DA neurons differentiation protocols**	**Manifestations in post-transplantation PD models**
ESCs	I. Co-culturing with feeder cells (SDIA) (Kawasaki et al., 2000; Zeng et al., 2004; Brederlau et al., 2006) II. Soluble growth factors/chemicals (Lee et al., 2000; Schulz et al., 2004; Takagi et al., 2005; Yan et al., 2005; Yang et al., 2008) III. genetic manipulations (Kim et al., 2002; Chung et al., 2005; Andersson et al., 2006) IV. Specific combinations of I, II, and III (Perrier et al., 2004; Park et al., 2005; Roy et al., 2006; Sonntag et al., 2007) V. Several morphogens (such as all-trans RA, SHH, FGF, EGF, BMPs, GDNF) facilitate neural lineage induction (Fraichard et al., 1995; Ciccolini and Svendsen, 1998; Guan et al., 2001; Buytaert-Hoefen et al., 2004; Perrier et al., 2004; Li et al., 2005)	1. DA phenotype 2. Extended neurite outgrowth 3. Synaptic marker 4. Considerable behavioral recovery (Kim et al., 2002; Schulz et al., 2004; Takagi et al., 2005; Yan et al., 2005; Brederlau et al., 2006; Yang et al., 2008; Takahashi et al., 2009; Kriks et al., 2011; Daadi et al., 2012)
NSCs/NPCs	I. Soluble neurotrophic factors (GDNF et al.) and cytokines (IL-1, IL-11, LIF) (Nishino et al., 2000; Yang et al., 2004; Christophersen et al., 2006; Harrower et al., 2006) II. Genetic modification factors (Nurr1, SHH, Bcl-XL, Mash1, Pitx3) (Liste et al., 2004, 2007; Park et al., 2006; O'Keeffe et al., 2008) III. TH^+^ neurons output can be further enhanced by low oxygen culturing condition (Studer et al., 2000)	1. Integration into nigrostriatal pathway 2. Restoration of SN-striatum projection 3. Resumption of DA synthesis and release 4. Relief of PD-like symptoms (Studer et al., 1998; Harrower et al., 2006; Park et al., 2006; Redmond et al., 2007; O'Keeffe et al., 2008)
MSCs	I. Chemical induction, growth factors, signaling molecules, co-culturing with feeder cells and employment of conditioned medium (Hermann et al., 2004; Long et al., 2005; Wang et al., 2005; Chu et al., 2006; Choong et al., 2007; Anghileri et al., 2008; Fu et al., 2008; Zhang et al., 2008; Barzilay et al., 2009; Trzaska et al., 2009; Datta et al., 2011) II. Genetic engineering of MSCs (by virtue of protein or gene delivery of neurotrophic factors, particularly GDNF, VEGF, and neurturin) (Rosenblad et al., 1999; Bjorklund et al., 2000; Kozlowski et al., 2000; Kordower et al., 2006; Gasmi et al., 2007; Herzog et al., 2007; Eberling et al., 2009; Johnston et al., 2009; Kells et al., 2010; Fierro et al., 2011)	1. Survival of grafted cells 2. Expression of TH 3. Obvious behavior recovery (Rosenblad et al., 1999; Bjorklund et al., 2000; Kozlowski et al., 2000; Woodbury et al., 2000; Kordower et al., 2006; Gasmi et al., 2007; Herzog et al., 2007; Offen et al., 2007; Ye et al., 2007; Bouchez et al., 2008; McCoy et al., 2008; Eberling et al., 2009; Johnston et al., 2009; Kells et al., 2010; Delcroix et al., 2011; Fierro et al., 2011; Xiong et al., 2011; Mathieu et al., 2012)

*All-trans RA, all-trans retinoic acid; BMPs, bone morphogentic proteins; SHH, sonic hedgehog; Nurr1, Nuclear receptor related 1 protein; Bcl-XL, B-cell lymphoma-extra large; Mash1, achaete-scute homologueiy ash1; Pitx3, Pituitary homeobox 3*.

### Current status and future prospects

Up to now, there have existed several demonstrations that DA-rich transplants derived from ESCs have generally maintained their DA-induced phenotype, extended neurite outgrowths, expressed synaptic markers and produced considerable behavioral recovery following grafting into the PD animal models (Kim et al., 2002; Takagi et al., 2005; Brederlau et al., 2006; Yang et al., 2008; Muramatsu et al., 2009; Takahashi et al., 2009; Kriks et al., 2011; Daadi et al., 2012; see Table 1). Similar to the outcomes in PD animal models, patients receive human fetal tissues transplantation demonstrate significant reduction in motor deficits with considerable graft survival, dopamine release and synaptic integration of DA neurons (Kordower et al., 1995; Piccini et al., 1999; Mendez et al., 2005; Barker et al., 2013; Petit et al., 2014). However, owing to limited tissue availability and other related issues, the employment of fetal tissue is unlikely to become a routine treatment for PD. To break through the dilemma, the established ESCs lineage induction protocols (see Figure 2) can produce theoretically limitless population of homogenous mesencephalon DA neurons, thus holding great promise for ESCs based CRT strategies. As is the case for all pluripotent stem cells, nevertheless, hESCs possess the potential concern of teratoma generation in the intended zone of transplantation. Besides, the graft ESCs may cross blood brain barrier, integrate into ectopic brain regions functionally and secret active factors to produce adverse effects on central nervous system (CNS). Moreover, allogeneic hES cells and their derivatives may induce immune rejection when grafted into the recipient's brain (Tomaskovic-Crook and Crook, 2011). Apart from that, the graft induced dystonia and dyskinesia is another tough nut to crack (Freed et al., 2001; Olanow et al., 2003). As for the concerns above, what can we do in future stem cell research? On the whole, it is imperative to remove or reduce remnant ESCs to safe levels from the final graft product by means of fluorescent activated cell sorting (FACS), magnetic activated cell sorting (MACS), mitotic retardation, and/or directed differentiation so as to reduce the tumorigenic hazards (Brederlau et al., 2006). In addition, an evaluation of the graft cell biodistribution, potential ectopic toxicity and understanding of the immune status of ESCs-derived cells and the CNS undergoing neurodegenerative insult will better guarantee the success of ESCs based CRT in PD. Regrettably, there are no, by far, US Food and Drug Administration (FDA)-approved ongoing clinical trials using hESC-derived cells for transplantation in PD patients as a result of the potential concern above. Whereas, given the fascinating intrinsic differentiation potencies and concerted efforts of researchers worldwide, the hESCs derived DA neurons will be bound to reach clinical stage and translated to clinical application.

## Neural stem cells (NSCs)

**Figure 3:**
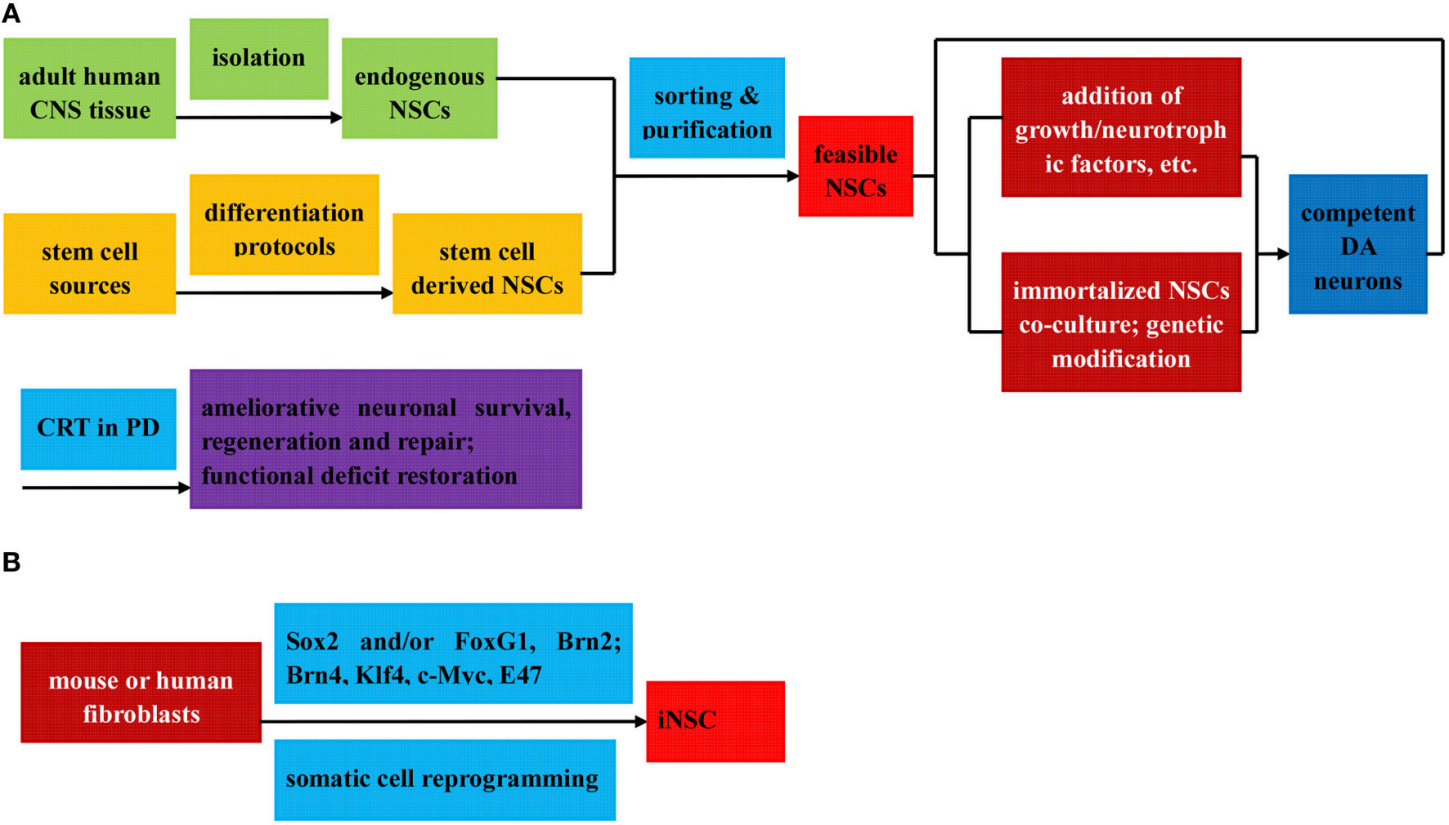
**Flow diagram of the preparation (direct isolation or induced differentiation) and respective application of NSCs in PD treatment**. The NSCs can be divided into three categories: (1) Endogenous NSCs, isolated directly from adult human CNS tissue **(A)**; (2) Stem cell derived NSCs, induced from various stem cells via diverse differentiation protocols **(A)**; (3) iNSC, reprogrammed from mouse or human fibroblast by Sox2, with or without other transcription factors **(B)**; all of which can be further sorted and purified to obtain feasible NSCs. Among others, the stem cell derived NSCs can be further induced to differentiated into DA neurons by means of two protocols below: (i) addition of growth factors, neurotrophic factors, cytokines, and et al. to culture medium to induce the neurosphere formation; (ii) co-culture with immortalized NSC lines to introduce genetic modification factors. Ameliorative neuronal survival state and restorative functional deficits can be observed when the feasible NSCs and competent DA neurons above are applied in CRT treatment of PD.

The discovery in the early 1990s of stem and progenitor cells in the adult mammalian CNS (Reynolds and Weiss, 1992) challenged the long-standing “no new neuron” doctrine attributed to the eminent scientist Ramony et al. opening the window to the potential of NSCs for CRT of PD. NSCs, a subtype of stem cells capable of self-renewing and generating the main phenotypes of the nervous system in both embryo and adult, are committed to the neural lineage differentiation and presumably form neurons, oligodendrocytes, and astrocytes *in vivo*. In case of physiological condition, the proliferation and self-renewal of NSCs sustain a delicate balance by means of dividing symmetrically to maintain their quantities and asymmetrically to give rise to multifarious differentiated progenies. There are miscellaneous origins of NSCs, which, by and large, can be classified into two types: endogenous NSCs and stem cell-derived NSCs. Endogenous NSCs exist throughout life and are mainly found in specific niches of brain such as subventricular zone (SVZ), subgranular zone (SGZ) of hippocampal dentate gyrus (DG), corpus striatum, olfactory bulb, and cortex (Davis and Temple, 1994; Gage et al., 1995; Gage, 2000; Smith et al., 2003), which are responsible for the regeneration of new neurons to restore the functions of the brain and spinal cord. While the latter such as ESCs, MSCs, and iPSCs can also serve as a source for NSCs production, holding a great promise for regeneration and restoration of the CNS. In summary, there exist two culture protocols to induce the differentiation of NSC lines: (1) add to brain tissue culture medium encompassing growth factors (FGF, EGF), neurotrophic factors (GDNF, BDNF), cytokines (ILs, LIF, and so on) and then form free-floating colonies (neurospheres); (2) co-culturing with immortalized NSC lines to introduce genetic modification factors(Bcl-XL, Nurr1, Mash1, and Pitx3; see Figure 3). Besides, it has been reported that Sox2, with or without other transcriptional factors, is capable of directly reprogramming mouse or human fibroblasts into a bran-new NSCs identity termed as iNSCs (Han et al., 2012; Lujan et al., 2012; Ring et al., 2012; see Figure 3).

PD, a neurodegenerative disease associated to aging and suggested to be a consequence of deficiency of NSCs pool in the affected brain regions, is an appropriate candidate for CRT. Therefore, the replacement of NSCs, either endogenous NSCs, iNSCs, or stem cell-derived neural stem cells, into impaired brain is highly expected as a possible therapeutic mean for PD (see Figure 1). After the successful establishment of neural stem/precursor/progenitor cells (NPCs/NSCs) of multifarious origins, a rapid, reliable, and long-term production of human NPCs would be of immense practical value to both neuroscientists and clinical neural transplantation trials. In terms of proliferation and differentiation of fetal NPCs, Studer et al. in 1998 demonstrated that NPCs from the rat VM could grow and expand in bFGF-embedded system and then differentiate to yield a significant number of TH+ neurons, accompanying functional recovery when transplanted into rat PD models (Studer et al., 1998). As a matter of fact, introduction and specific combination of soluble factors, neurotrophic factors and genetic modification factors have enhanced the production of DA neurons as well (Sanchez-Pernaute et al., 2001; Storch et al., 2001; Liste et al., 2004, 2007; Yang et al., 2004; Christophersen et al., 2006). Since then, a number of studies explicitly exploring the formation of DA neurons from NPCs have been attempted with the introduction of either soluble factors such as interleukin-1 (IL-1), IL-11, leukemia inhibitory factor (LIF), and GDNF (Nishino et al., 2000; Sawamoto et al., 2001; Harrower et al., 2006; Timmer et al., 2006) or genetic modification factors involved in DA neurons specification and survival (such as Nurr1, SHH and Bcl-XL; Nurr1 and Mash1 or Pitx3; Park et al., 2006; O'Keeffe et al., 2008), resulting in remarkable DA differentiation and significant functional recovery on grafting into PD animal models (Nishino et al., 2000; Sawamoto et al., 2001; Harrower et al., 2006; Park et al., 2006; Timmer et al., 2006; Redmond et al., 2007; O'Keeffe et al., 2008; see Table 1). Additionally, the proportion of TH+ neurons could be further enhanced by culturing the cells in low oxygen conditions (Studer et al., 2000), and studies involving hNPCs have demonstrated similar outcomes as well, but with low percentage of TH+ DA neurons (Svendsen et al., 1997).

### Current status and future prospects

Full scale evaluation of the NSCs grafted PD models demonstrates that NSCs can integrate into nigrostriatal pathway, restore the projection of substantia nigra to striatum, resume DA synthesis and release and relieve PD-like symptoms (Studer et al., 1998; Harrower et al., 2006; Park et al., 2006; Redmond et al., 2007; O'Keeffe et al., 2008; see Table 1). Given the condition that NSCs possess unique capacity to expand and potential to differentiate into variously “wanted” neurons and glias, it seems that NSCs can be a perfect therapeutic candidate for neurological diseases, especially PD. Moreover, compared to the approach of generating DA neurons from an ESC source, the employment of NPCs is of obvious advantage as a result of its decreased tumorigenic potential and immunological rejections. However, it is difficult, as a matter of fact, to secure enough adult human CNS tissues for preparation of adult NSCs. It is imperative, for this reason, that a stable and homogeneous human NSC lines should be established to serve as an ideal alternative cellular source.

In future NSCs based CRT studies, the obstacles below should be overcome so as to enable NSCs be translated to PD therapy: (1) generating specific cell types of neurons or glia suitable for cellular grafts in great quantity from NSCs; (2) erasing safety concerns related to tumor formation, immunological rejection and biodistribution related toxicity following NSC transplantation; (3) exploring the implicit mechanism that enable the NSCs mediated functionally recovery. Continued and extensive progress in stem cell research in both basic and pre-clinical settings will prompt the NSCs-based therapy in neurodegenerative diseases, especially in PD.

## Mesenchymal stem cells (MSCs)

MSCs, a non-haematopoietic, multipotent subtype cell lines arising mainly from the stromal structures of the bone marrow (Prockop, 1997) other than adipose tissue (Schaffler and Buchler, 2007), umbilical cord (Fu et al., 2006), dermis (Kuroda et al., 2011), and peripheral blood (Kim et al., 2006), generally differentiating into osteocytes, chondrocytes and adipocytes *in vivo* (Bianco et al., 2001; see Figure 1). Apart from the regular differentiation subclasses, MSCs possess the trans-differentiation potential to form other non-mesenchymal cell types (Jiang et al., 2002), especially the neurogenic potential to trans-differentiate into nestin-positive neurospheres in the presence of EGF and bFGF (Kim et al., 2006). Furthermore, MSCs with neurogenic potential (neurally-induced MSCs), especially the MSCs derived from the accessible source of peripheral blood, may represent a wholly new source of cells for autologous transplantation therapies in neurodegenerative disease. As for PD, due to the focused loss of DA neurons, it is a particularly suitable candidate for MSC mediated CRT.

**Figure 4:**
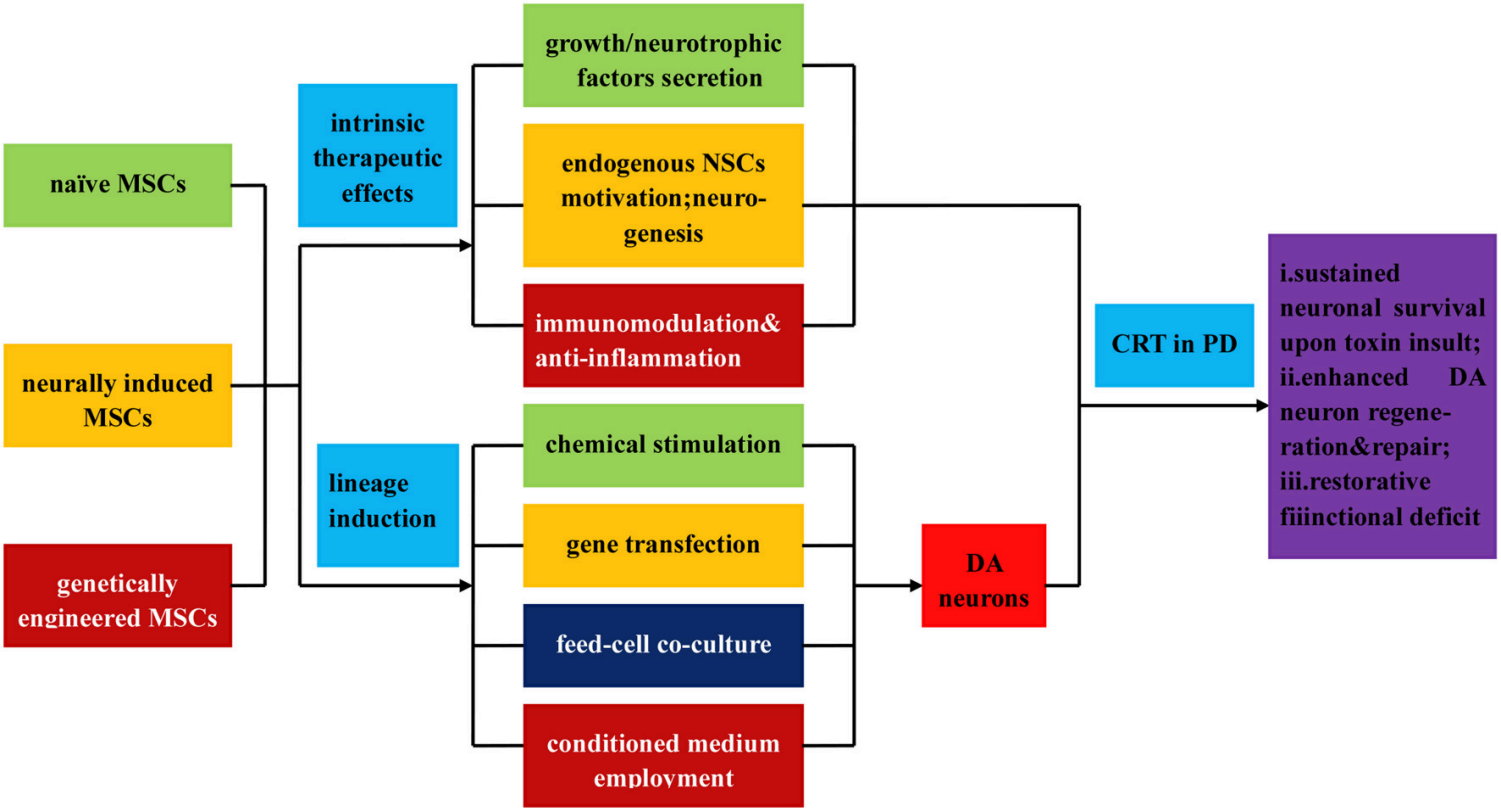
**Illustration of the direct isolation, induced differentiation, and respective application of MSCs in PD treatment**. MSCs can be classified into three types: naïve MSCs, neurally induced MSCs and genetically engineered MSCs, all of which can directly exert intrinsic therapeutic effects upon transplantation, such as (i) secretion of neutrophic factors, growth factors, and et al.; (ii) endogenous MSCs motivation, neurogenesis, and angionesis; (iii) immunomodulation and anti-inflammatory effects. In addition, MSCs can be inductively differentiated into DA neurons by virtue of (i) chemical stimulation; (ii) gene transfection; (iii) feed cell co-culture; (iv) employment of conditioned culture medium. Upon transplantation, the MSCs and differentiated DA neurons demonstrate (a) sustained neuronal survival upon toxin insult; (b) enhanced DA neurons regeneration and repair; (c) functional deficits restoration.

In general, there are two categories of MSCs: naive MSCs (directly isolated from mesenchymal tissues) and neurally-induced MSCs, which all hold immense promise for PD treatment (see Figure 4). MSCs isolated from different tissues can adopt morphological and phenotypical characteristics of neuronal cells under various culture conditions, part of which have been transplanted into PD models with observed functional recovery. Among them, Naive bone MSCs (BMSCs) and neurally-induced bone MSCs (BMSCs) all have been tested for therapeutic effects in PD models by several groups, both demonstrating survival of grafted cells, expression of TH and obvious behavior recovery (Woodbury et al., 2000; Offen et al., 2007; Ye et al., 2007; Bouchez et al., 2008; Delcroix et al., 2011), but restoration effects of neurally-induced BMSCs seem to be more pronounced (Woodbury et al., 2000). Besides, MSCs isolated from adipose tissue and umbilical cord have equally shown beneficial effects in PD models as well (McCoy et al., 2008; Xiong et al., 2011; Mathieu et al., 2012). Moreover, genetically engineered MSCs have also been demonstrated to exhibit therapeutic potential in PD treatment. Numerous studies have demonstrated that protein or gene delivery of growth factors, particularly GDNF, VEGF and neurturin, effectively protects DA neurons in a succession of rodent and primate models of PD (Rosenblad et al., 1999; Bjorklund et al., 2000; Kozlowski et al., 2000; Kordower et al., 2006; Gasmi et al., 2007; Herzog et al., 2007; Eberling et al., 2009; Johnston et al., 2009; Kells et al., 2010; Fierro et al., 2011; see Table 1).

Similar to ESCs induction, most MSCs induction protocols demand different combinations of chemicals, growth factors and signaling molecules (Long et al., 2005; Wang et al., 2005; Chu et al., 2006; Choong et al., 2007; Anghileri et al., 2008; Fu et al., 2008). In summary, differentiation of MSCs into DA neurons can be achieved through different protocols based on chemical induction, gene transfection, co-culturing with glial, neuronal and neuronal stem cells, and employment of conditioned medium (Hermann et al., 2004; Zhang et al., 2008; Barzilay et al., 2009; Trzaska et al., 2009; Datta et al., 2011; see Figure 4). To date, there are several possible underlying mechanisms below interpreting the therapeutic effects of MSCs for PD (see **Figure 6**).

1. Secretion of various growth factors, cytokines, extra cellular matrix (ECM) proteins, and potent neuro-regulatory molecules to create a favorable environment for neural regeneration.
  MSCs have been reported to secrete an array of growth factors and cytokines, including BDNF, nerve growth factor (NGF), GDNF, vascular endothelial growth factor (VEGF), neurotrophin-3 (NT-3) and stromal cell-derived factor-1 (SDF-1; Arnhold et al., 2006; Crigler et al., 2006; Croitoru-Lamoury et al., 2007; Pisati et al., 2007; Park H. J. et al., 2008; Wilkins et al., 2009; Blandini et al., 2010; Jiang et al., 2010; Kim et al., 2010; Lattanzi et al., 2011). It is presumable that the growth factors secreted from grafted MSCs and stimulated host cells are implicated in the therapeutic effects of MSCs in different animal models of PD (Crigler et al., 2006; Park H. J. et al., 2008; Blandini et al., 2010). Besides, MSCs can produce extracellular matrix (ECM) proteins that can support neural cell attachment, growth, neuritogenesis, and functional restoration (Aizman et al., 2009; Lai et al., 2010). Therefore, the secretion of multiple paracine factors by MSCs is involved in DA protection and repair, which may partially account for the therapeutic effects of MSCs for PD.
2. Activation of endogenous restoration mechanisms to facilitate neurogenesis, angiogenesis and decrease the loss of DA neurons (anti-apoptosis).
  *In vitro* studies have shown that transplantation of MSCs can stimulate proliferation, migration and differentiation of the endogenous NSCs (Munoz et al., 2005; Bai et al., 2007; Robinson et al., 2011). In addition, MSCs can exert an influence on endogenous NSCs indirectly through stimulation of astrocytes to secrete growth factors, such as BDNF and NGF, which promote neurogenesis (Song et al., 2002; Munoz et al., 2005). In addition, another study has shown that transplantation of human MSCs to an MPTP mouse model of PD augments neurogenesis both in SVZ and SN and increases differentiation of NSCs toward DA neurons, suggesting effects achieved through EGF secretion and an increased expression of EGF receptor in the SVZ (Park et al., 2012a). Another significant feature of the regenerative effects of MSCs is their ability to promote endothelial cell proliferation and angiogenesis, since angiogenesis and neurogenesis are coupled processes (Kinnaird et al., 2004a,b; Teng et al., 2008; Park et al., 2012a). In a word, through release of paracrine factors, MSCs can exert an effect on the host tissue to facilitate intrinsic restorative processes such as neurogenesis and angiogenesis and decrease loss of DA neurons simultaneously.
3. Immunomodulation and anti-inflammatory effect.
  There are enormous evidences showing that extensive proliferation of activated microglia has been observed postmortem in the SN of PD patients (McGeer et al., 1988; Langston et al., 1999). Moreover, elevated levels of pro-inflammatory cytokines, such as tumor necrosis factor (TNF), interleukin-1 beta (IL-1 β), and interferon-gamma (IFN-γ) have been detected in brains of PD patient (Hunot et al., 1999; Nagatsu et al., 2000). Additionally, it has been shown that immunosuppressant cyclosporin A attenuates DA degeneration in PD animal models (Kitamura et al., 1994). Together these studies illustrate the importance of MSCs immunomodulatory and anti-inflammatory effects in developing treatments for PD.

### Current status and future prospects

As a promising restorative therapeutic alternative, stem cell therapy has come to the forefront of the PD research field as a promising regenerative therapy, especially in relation to MSCs. On the one hand, MSCs can be easily procured and expanded, without the use of other supportive cells. On the other, MSCs are not burdened with the ethical and immunorejection issues associated with ESCs. Regrettably, the number of observed MSCs-derived neurons and glial cells is rather small and fail to restore a normal cyto-architecture, though several *in vivo* studies have demonstrated MSCs are capable of differentiating into neuronal cells following graft (Mimura et al., 2005; Lu et al., 2006; Alexanian et al., 2008). Therefore, how to generate sufficient and specific subtype of neurons or glia suitable for cellular grafts in future study is a primary knot to be straightened out. As is the case for ESCs and NSCs, safety concerns related to tumor formation, immunological rejection and biodistribution related toxicity should be resolved as well so as to translate MSCs into PD therapeutic options eventually. We are convinced that MSCs hold great promise to be a member of stem cell pool available for CRT of PD, despite MSC research is still in the start-up phase of clinical pipeline.

## Induced pluripotent stem cells (iPSCs)

In 2006, Yamanaka et al. have shown that ESC-like cells could be derived from embryonic and adult mouse fibroblasts by means of retrovirally introducing OSKM (Oct3/4, Sox2, Klf4, and c-Myc) gene transcription factors (Takahashi and Yamanaka, 2006), latterly awarded Nobel Prize for “the discovery that mature somatic cells can be reprogrammed to reenter into pluripotency.” The ESC-like pluripotent cells obtained from reprogramming procedure are coined as iPSCs (see Figure 1). Interestingly, iPSCs share similar properties to ESCs in morphology, proliferation, expression of specific ESCs marker genes, embryoid bodies (EBs) formation *in vitro*, teratoma formation *in vivo*, and tripotent differentiation into three germ layers (Rodolfa and Eggan, 2006; Takahashi and Yamanaka, 2006). However, what is frustrating is that these iPSCs, derived from non-human origins, occupy a different global gene expression profiles from ESCs and fail to produce adult chimeric mice. There has been a substantial lack of iPSCs that directly reflects the genetic and physiological uniqueness of the human condition until the maiden prominent induction of iPSCs from human somatic cells accomplished by two different set of transcription factors (Takahashi et al., 2007; Yu et al., 2007). Since then, many research groups have successfully established iPSCs from various types of human somatic cells (Park I. H. et al., 2008; Hanna et al., 2010), further demonstrating that epigenetic mechanism is implicated in the reprogramming process of many, if not all, types of somatic cells.

For PD research in particular, the iPSC technology enables the generation of sufficient physiologically relevant, patient-specific midbrain DA neurons, which may be highly valuable for basic research on the molecular and cellular mechanisms of PD, for drug discovery research to identify disease-modifying therapies, and for cell-based therapy utilizing autologous donor materials (Pu et al., 2012). Besides, the PD patient-specific iPSCs (PD-iPSCs) offer the possibility for autologous transplant, which would significantly reduce graft rejection. However, enormous concerns culminate for the employment of integrating viruses that probably intrinsically modify the genome of host cells, especially the introduction of integrated oncogenes such as c-Myc and Klf4. Therefore, new reprogramming methods such as reduction of the integrating transcription factors, especially the oncogenic c-Myc (Nakagawa et al., 2008) and application of non-integrating viruses (Stadtfeld et al., 2008; Zhou and Freed, 2009; Ban et al., 2011) have been employed to circumvent the viral integration events. In addition, several non-viral delivery vectors such as expressing plasmids (Okita et al., 2008, 2010; Hartung et al., 2013), episomal vectors (Yu et al., 2009; Choi et al., 2011; Chou et al., 2011; Okita et al., 2011; Wang et al., 2011; Dowey et al., 2012), minicircle vectors (Jia et al., 2010), piggyBac transposon (Woltjen et al., 2009; Tsukiyama et al., 2011; Kues et al., 2013), synthetic modified RNAs (Warren et al., 2010; Anokye-Danso et al., 2011; Miyoshi et al., 2011; Yoshioka et al., 2013), protein (Kim et al., 2009; Zhou et al., 2009; Cho et al., 2010) and small molecules (Huangfu et al., 2008; Shi et al., 2008; Li Y. et al., 2011; Hou et al., 2013) have also successfully been used to established integration free iPSCs (see Table 2). Compared with other types of stem cells, iPSCs appear to be a more preferential CRT candidate by virtue of more superior pluripotency, more distinctive differentiation features and more accessible sources. The preparation, application, current status, potential challenges, and future prospects of the multifarious iPSCs are further elaborated in the text below.

**Table 2:** **Constellation of multifarious delivery methods to induce iPSCs**.

**Delivery methods**	**Vectors**	**Crews**	**References**
Integrating viral delivery	Retrovirus	OSKM	Takahashi and Yamanaka, 2006
		Oct4, Sox2, VPA	Huangfu et al., 2008
	Lentivirus	ONSL	Yu et al., 2007
Non-integrating viral delivery	Non-integrating adenovirus	OSKM	Stadtfeld et al., 2008
			Zhou and Freed, 2009
	Temperature–sensitive Sendai Virus	OSKM	Fusaki et al., 2009
		OSKM	Seki et al., 2010
		OSKM	Ban et al., 2011
		OSKM	Nishishita et al., 2012
	Expressing plasmids	OSKM	Okita et al., 2008
		OSKM	Okita et al., 2010
		OSKM	Hartung et al., 2013
	Episomal vectors(oriP/EBNA1)	OSKM, Nanog, Lin28	Yu et al., 2009
		SV40 larger T gene	
		OSKM, Lin28	Choi et al., 2011
			Chou et al., 2011
			Dowey et al., 2012
		Oct4, Sox2, Klf4, Nanog	Wang et al., 2011
		Oct4, Sox2, Klf4, L-Myc	Okita et al., 2011
	Minicircle vectors	ONSL	Jia et al., 2010
	Polycistronic Vectors/Cre-loxP System	OSKM	Kaji et al., 2009
		OSKM	Karow et al., 2011
		OSKM, picornaviral 2A peptide	Loh et al., 2012
	Transposon		
	piggyBAC	OSKM	Woltjen et al., 2009
	piggyBAC	OSKM	Tsukiyama et al., 2011
	Sleeping beauty	OSKM	Kues et al., 2013
Non-viral delivery	RNAs		
	mRNA	OSKM	Warren et al., 2010
		Oct4, Sox2, Klf4, c-Myc, or GLIS1	Yoshioka et al., 2013
	miRNA	miRNA302/367cluster	Anokye-Danso et al., 2011
		miRNAfamily(miRNA200c, miRNA302s, miRNA369s)	Miyoshi et al., 2011
	Protein	CPP, OSKM, VPA	Zhou et al., 2009
		CPP,OSKM	Kim et al., 2009
		ESC-derived proteins	Cho et al., 2010
	Small molecules	VPA, Oct4, Sox2	Huangfu et al., 2008
		Oct4, Klf4, BIX-01294	Shi et al., 2008
		Oct4, VPA, CHIR99021, 616452	Li Y. et al., 2011
		Wholly small-molecule compounds	Hou et al., 2013

### Integrating virus mediated induced pluripotent stem cells (iPSCs)

Since the initial astonishing establishment of iPSCs by enforced expression of the OSKM transcription factors mediated by retroviral integration (Takahashi and Yamanaka, 2006), emerging studies have relied on viral vectors, such as adenovirus (Zhou and Freed, 2009), lentivirus (Yu et al., 2007; Fierro et al., 2011) and retrovirus (Huangfu et al., 2008), to deliver transcription factors into target cells aiming to reprogramme to pluripotent state for subsequent lineage specification. No matter Yamanaka's OSKM reprogramming formula, Yu's ONSL (Oct4, Nanog, Sox2, and Lin28), recombination recipe or other versatile combinations, iPSCs have been proving to exhibit pluripotency at levels similar to ESCs. And a recent study indicates that OSKM and ONSL cocktails can act synergistically to reprogramme human somatic cells into iPSCs (Jung et al., 2014). Although highly efficient lineage trans-differentiation and long-term expression can be achieved with viral vectors, however, iPSCs technology is complicated by the potential risks posed by genome-integrating viruses, which are randomly but permanently integrated into the host genome at multiple sites together with viral vector backbone. Such genome-integrating viral vectors can produce insertional mutations which may influence differentiation potential, or even result in tumorigenesis especially due to reactivation of the c-Myc oncogene (Thomas et al., 2003; Okita et al., 2007). Besides, viral delivery is also plagued by limited cargo capacity, resistance to repeated infection, complicated operation procedures and unbridled genome alterations (Thomas et al., 2003; Park et al., 2012b). Therefore, alternative ways such as non-integrating viral and non-viral reprogramming methods have been developed to circumvent these risks and drawbacks.

### Non-integrating virus mediated induced pluripotent stemcells (iPSCs)

Although mouse and human somatic cells have been successfully reprogrammed into iPSCs by viral delivery of specific and versatile cocktail of transcription factors and chemicals (small molecules), the incorporation of viral DNA into host genome may lead to disruption of gene transcription and malignant transformation, posing as a serious safety concern. To proceed with iPSCs for PD research and therapy, non-integrating viral vectors have been proposed as alternatives. Replication-defective adenovirus has been proven to be a perfect candidate of viral vector to enable the insertion of reprogramming genes into somatic cells without integration into the host chromosomal DNA (Graham and Prevec, 1992; He et al., 1998). Moreover, the transient gene expression, which persists for several days, can provide sufficient time to reprogram fibroblasts into pluripotent cells. Stadtfeld et al. have used adenoviral vectors to establish mouse iPSCs without viral integration from liver cells and fibroblasts (Stadtfeld et al., 2008). Following his pioneering work, Zhou et al. has also successfully generated human iPSCs from human embryonic fibroblasts employing non-integrating adenoviral vectors expressing the OSKM transcription factors. Southern blots and polymerase chain reaction (PCR) have demonstrated that there does not display viral DNA integration; fingerprinting and karyotype analysis have confirmed that these iPSCs are derived from the parent human fibroblasts. Additionally, the established iPSCs are capable of differentiating into all three germ layers *in vitro*, including DA neurons (Zhou and Freed, 2009). Since Sendai Virus (SeV), a negative sense single-stranded RNA virus residing in the cytoplasm of infected cells, does not integrate into the host genome, moreover, readily removed by Ab-mediated negative selection, Fusaki and Li et al. have respectively reported efficient establishment of transgene-free induced pluripotent stem cell lines employing a vector based on Sendai virus without host genome integration (Li et al., 2000; Fusaki et al., 2009). Another defined method for the generation of iPSCs by the employment of non-integrating temperature-sensitive Sendai Virus Vector (SeV TS7) from a single subcloning in the naive state under feeder-free conditions, which solves some of the safety concerns related to use of xeno- or allogeneic-material in culture, and contribute to the characterization and the standardization of iPS cells intended for use in a clinical setting (Nishishita et al., 2012). Furthermore, human terminally differentiated circulating T cells (hTDCTCs) have also been reprogrammed into iPSCs with mediation of temperature-sensitive mutated SeV vector which can reduce transgene expression and SeV residue in generated cell lines (Seki et al., 2010; Ban et al., 2011). In summary, the non-integrating viruses such as adenovirus (Stadtfeld et al., 2008; Zhou and Freed, 2009; Ban et al., 2011) and Sendai virus (Li et al., 2000; Fusaki et al., 2009; Seki et al., 2010; Ban et al., 2011; Nishishita et al., 2012) have been regarded as ideal vectors for generating iPSCs without exogenous gene integration. However, iPSCs research is entering an age dedicated to epigenetic reprogramming without genetic modification. Therefore, the development of non-viral integration-free iPSCs is a necessity.

### Non-viral vector induced pluripotent stem cells (iPSCs)

Despite the imposed viruses are silenced or removed and in turn the endogenous genes encoding reprogramming transcription factors are activated during the process of viral vector mediated iPSC generation, the utilization of integrating or non-integrating viruses still raises several potential safety concerns, known or unknown, which restrict the further clinical application of this technique. Therefore, non-viral vector induced pluripotent stem cells (iPSCs) have been exploited as alternative methods.

#### Expressing plasmids

Expressing plasmids have been explored as a viral vector substitute to attempt to establish transgene free iPSCs, the first alternative to be investigated among the enormous classes of non-viral vectors. Repeated transfection of mouse embryonic fibroblasts (MEFs) with one expression plasmids containing the complementary DNAs (cDNAs) of Oct3/4, Sox2, and Klf4 and the other one involving the c-Myc cDNA, has enabled generation of non-plasmid integration iPSCs, which could produce teratomas when transplanted into mice and contributed to adult chimeras, but with a reprogramming efficiency falling far below that of viral vectors (Okita et al., 2008, 2010). Besides, a more recent study employing transient transfection approach with conventional expression plasmids has successfully established iPSCs as well, eliminating the risk of insertional mutagenesis and providing a straightforward platform for further optimizations and translation to other lineages (Hartung et al., 2013). Although the plasmid mediated iPSCs preparation method could tremendously reduce the host genome integration risk, the unsatisfactory reprogramming efficiency needs to be refined before its further clinical application for PD treatment.

#### Episomal vectors

Episomal iPSC reprogramming vectors are a bran-new non-viral vectors designed to provide the optimal delivery system for generating transgene-free and virus-free iPSCs in a feeder-free culture system. Derived from the Epstein-Barr (EB) virus, oriP/EBNA1 (Epstein-Barr nuclear antigen-1) based vector, involving three plasmids expressing seven reprogramming factors (Oct4, Sox2, c-Myc, Klf4, Nanog, Lin28, and SV40 large T gene), is a perfect candidate for introducing reprogramming factors into human somatic cells, as these plasmids can be transfected without the need for viral packaging, and can be subsequently removed from cells by culturing in the absence of drug selection. It is originally developed by Yu et al. to establish human iPSCs from human foreskin fibroblasts by the employment of oriP/EBNA1 based vector, and further genome analysis detects no episomal vector integration in the host genome but with a iPSCs yield far below that of viral vectors (Yu et al., 2009). Choi et al. have shown that fibroblasts and EBV (Epstein-Barr virus)-immortalized B cell lines derived from multiple inherited disease patient could be reprogrammed into iPSCs via single transfection of EBNA-1/OriP based episomal vector, with no integration of the reprogramming-related transgenes or the EBV-associated genes (Choi et al., 2011). Following these studies, several research groups have established iPSCs employing the oriP/EBNA1 based vector, but with fewer reprogramming factors, especially the absence of c-Myc (Wang et al., 2011), and higher reprogramming efficiency (Chou et al., 2011; Okita et al., 2011; Dowey et al., 2012). These studies demonstrate that the establishment of hiPSCs does not require genomic integration or the continued exposure to exogenous reprogramming factors, which move one step forward to the clinical application of human iPSCs for PD treatment.

#### Minicircle vectors

Minicircle vectors, supercoiled DNA molecules lacking a bacterial replication origin and antibiotic resistance markers, are predominantly composed of eukaryotic expression profiles, possessing higher transfection efficiencies and more sustained ectopic expression compared to plasmids as a result of its lower activation of exogenous silencing mechanisms (Chen et al., 2003, 2005), thus may representing an ideal mechanism for generating iPSCs. Jia et al. have reported to obtain hiPSCs from human adipose stem cells (hASCs) by means of a minicircle vector, encompassing a single cassette of ONSL reprogramming factors plus a green fluorescent protein (GFP) reporter gene, yielding a reprogramming efficiency (~0.005%) similar to that (~0.01%) of viral-based method (Jia et al., 2010). The minicircle vector delivery method, generating transgene-free iPSCs from adult donor sources, and requiring only a single vector without the need for subsequent drug selection, vector excision, or inclusion of oncogenes such as c-Myc and SV40, is ideally suited for facilitating iPSCs application in PD treatment.

#### Polycistronic vectors/Cre-loxP system

A non-viral transfection of multiple transcription factors expressing polycistronic vectors combined with subsequent site-specific Cre-recombinase excision to remove the reprogramming cassette has been employed to generate iPSCs without transgene integration. With the preparation protocols described above, iPSCs free of exogenous reprogramming factors have been secured via ensuing Cre-recombinase treatment from murine and human fibroblasts (Kaji et al., 2009; Karow et al., 2011). Moreover, it has been reported that, after the reprogramming procedure identical to the studies above have been accomplished, the reprogramming cassette can also be removed using mRNA transfection of Cre recombinase (Loh et al., 2012). In relation to PD, direct reprogramming of viral reprogramming factor-free iPSCs have been demonstrated to be generated from patients with idiopathic PD via Cre-recombinase excisable viral constructs. In addition, these PD specific iPSCs can further differentiate into DA neurons (Soldner et al., 2009). Although the obvious merits implicit in the iPSCs preparation methods above, insertional mutation concerns resulting from residual vector sequences still remain.

#### Transposons

The piggyBAC (PB) transposon is a mobile genetic element efficiently transposing between vectors and chromosomes by means of “cut and paste” mechanism. Inverted Terminal Repeats (ITRs) derived from the PB transposon are used to flank a transgene with recognition sequences for a transposase enzyme. Insertions and excisions can then be triggered by regulated, transient expression of the transposase. On the basis of the principles above, the non-viral piggyBac transposon and transposase system have been originally employed by Woltjen et al. to reprogram murine and human embryonic fibroblasts into iPSCs, from which the reprogramming factors are then removed from established iPSCs using transposase-stimulated piggyBac excision mechanism (Woltjen et al., 2009). Following this study, Dox-inducible system has been employed to remove the transgene expression, establishing iPSCs without exogenous gene expression (Tsukiyama et al., 2011). More recently, another type of transposon system, the Sleeping Beauty, has been used to deliver the transcription factors, thus enabling iPSCs establishment from fibroblasts (Kues et al., 2013). In accordance with expressing plasmids, episomal vectors, minicircle vectors, and Polycistronic Vectors/Cre-loxP System, the low reprogramming efficiencies and residual vector sequences may restrict its further clinical application for PD treatment.

#### RNAs (mRNA and miRNA)

RNAs, such as mRNA and miRNA, have been demonstrated, as a non-viral delivery system of transcription factors, to be capable of driving induction of pluripotency from terminally differentiated somatic cells. In 2010, Warren et al. demonstrated that repeated administration of synthetic mRNAs, incorporating modifications designed to bypass innate anti-viral responses, could reprogram differentiated human cells to pluripotency with conversion efficiencies and kinetics substantially surpassing established viral protocols (Warren et al., 2010). Besides, this simple, non-mutagenic, and highly controllable technology possess a broad reprogramming spectrum, capable of reprogramming multiple human cell types to pluripotency (Warren et al., 2010). Moreover, additional transfection of Lin28-encoding synthetic modified mRNA under hypoxic condition has been proven to facilitate reprogramming to iPSCs, with a much higher reprogramming efficiency and much shorter time period (Warren et al., 2010). What is interesting is that innate immune suppression enables frequent transfection with protein-encoding RNA, which may represent a versatile tool for investigating expression dynamics and protein interactions by enabling precise control over levels and timing of protein expression (Angel and Yanik, 2010). Recently, Yoshioka et al. has reported a simple but highly reproducible RNA-based iPSCs generation approach utilizing a single, synthetic self-replicating VEE-RF (Venezuelan Equine Encephalitis Encephalitis—reprogramming factor) RNA replicon that expresses four reprogramming factors, OCT4, KLF4, SOX2 with c-MYC or GLIS1(Glis Family Zinc Finger 1) at consistent high levels prior to regulated RNA degradation (Yoshioka et al., 2013). The VEE replicon, a positive-sense, single stranded RNA mimicking cellular mRNA with a 5′-Cap and poly(A) tail without encompassing a DNA intermediate, can be selectively retained or removed from the cell, so there does not exist a potential for genomic integration (Kinney et al., 1989). Therefore, the non-DNA and non-integrating, self-replicating VEE RNA approach has the potential to simplify the generation of human iPSCs for application in disease modeling and CRT for PD.

MicroRNAs (miRNAs) are a class of 18–24 nucleotides single stranded RNAs associated with a protein complex called the RNA-induced silencing complex, which has been found to function in numerous important processes in recent years. miRNAs are emerging critical regulators of cell function that frequently reside in clusters throughout the genome. In the wake of Warren et al.'s pioneering contribution, direct transfection of miRNAs has been demonstrated to be capable of reprogramming terminally differentiated somatic cells to pluripotent state. Anokye-Danso et al. have also shown that expression of the miR302/367 cluster can be capable of rapidly and efficiently reprogramming mouse and human somatic cells to pluripotency without a requirement for exogenous transcription factors. Besides, miRNA-based reprogramming approach is two orders of magnitude more efficient than standard OSKM-mediated methods (Anokye-Danso et al., 2011). Moreover, Miyoshi et al. indicate that it is possible to reprogram mouse and human cells to pluripotency by direct transfection of a combination of miR-200c plus miR-302s and miR-369s family miRNAs without vector-based gene transfer (Miyoshi et al., 2011), which holds significant potential for CRT of PD treatment. Besides, miRNAs have been proven to regulate iPSCs generation, as knock-down of key microRNA pathway proteins, in contrast, result in significant decreases in reprogramming efficiency (Li Z. et al., 2011). In fact, miRNAs influence a myriad of cell functions, implicit in the process of iPSCs establishment, starting from somatic cell isolation to iPSCs induction to colony derivation and characterization (Liao et al., 2011; Yang et al., 2011; Henzler et al., 2013; Dang and Rana, 2016). In transcription factor-induced reprogramming, it is speculated that miRNAs function in feedback loops with transcription factors and represent a key mechanism for fine-tuning gene expression. However, in-depth analyses of miRNA expression changes during reprogramming at the level of deep sequencing need to be further elaborated, which may hold enormous promise for PD research and therapy.

#### Proteins

Alternative iPSCs preparation methods to directly deliver reprogramming proteins rather than genetic materials or potentially mutagenic molecules into target cells, thus avoid introducing exogenous genetic modifications to target cells, have been established. However, a major hurdle for intracellular delivery of macromolecules such as proteins and exogenous genes is their limited ability to transverse the cellular membrane which are developed to ensure genetic diversity and for the protection from disadvantageous alien genes (Belting et al., 2005). Previous studies have demonstrated that various proteins can be delivered into cells *in vitro* and *in vivo* by means of conjugating them with a short peptide that mediates protein transduction, such as HIV tat and poly-arginine (Wadia and Dowdy, 2002; Michiue et al., 2005; Inoue et al., 2006), processing inclusion body proteins in E. coli with various solubilization and refolding techniques (Lafevre-Bernt et al., 2008), thus enabling facile and large-scale production of therapeutic proteins. Just as reported in Zhou et al.'s study, to generate recombinant proteins that could penetrate the plasma membrane of somatic cells, a poly-arginine (i.e., 11R) protein transduction domain has been designed and fused to the C terminus of the classic OSKM reprogramming factors. When the cell penetrating peptide (CPP)—OSKM reprogramming proteins complex being added to the cell culture system, the recombinant transcription factors readily enter cells and translocate to the nucleus. After four repeated protein transduction cycles, combined with the addition of valproic acid (VPA), a histone deacetylase inhibitor, iPSCs can be obtained with significantly improved reprogramming efficiency (Zhou et al., 2009). Likewise, Kim et al. has established the human protein-based iPSCs (piPSCs) by means of repeated transduction of OSKM reprogramming proteins, fused with a CPP, but without the alignment of VPA (Kim et al., 2009). On the whole, the DNA vector-free, direct protein transduction system described here eliminates limitations caused by viral or any other DNA-based reprogramming methods. However, the generation of human piPSCs is very slow and inefficient, in particular, the further purification of the protein involved requires further optimization. More recently, a single transfer of ESC-derived proteins into primarily cultured adult mouse fibroblasts, rather than repeated transfer or prolonged exposure to materials, has also been demonstrated to achieve iPSCs without the forced expression of ectopic transgenes. During the process, gene expression and epigenetic status were converted from somatic to ES-equivalent status, but little is known about the molecular mechanisms underlying reprogramming process by cellular proteins (Cho et al., 2010). The approach above is relatively simple and reproducible and does not require repeated transfer or prolonged exposure to materials or a combinatorial approach involving proteins and chemicals, which may could be further developed to provide tailored or patient-specific CRT for PD treatment.

#### Small molecules

Another way to avoid viral integration during iPSC generation is to employ chemicals or small molecules which are non-immunogenic and can be more easily administrated and standardized. Moreover, their effects on specific substrates are often reversible and dose-dependent. Small molecule libraries and combinations of compounds have been screened to identify substitutes to replace one or more reprogramming factors during iPSCs generation. For example, VPA, a histone deacetylase inhibitor, has been demonstrated to be capable of enabling reprogramming of primary human fibroblasts with only two factors, Oct4 and Sox2, without the need of the oncogenes c-Myc or Klf4 (Huangfu et al., 2008). Besides, another study has affirmed that the small molecule BIX-01294 (BIX), an inhibitor of the G9a histone methyltransferase, can improve the reprogramming efficiency in NPCs transduced with Oct3/4-Klf4 to a level comparable to transduction with the classic OSKM transcription factors. Thus, in this particular setting, this single small molecule, BIX, has been proven to be able to functionally replace viral transduction with c-Myc and Sox2 (Shi et al., 2008). Moreover, a specific chemical combination of VPA, CHIR99021 (a GSK3-β inhibitor), and 616452 (a TGF-β inhibitor, also termed VC6) have been identified to be sufficiently reprogramming from mouse embryonic and adult fibroblasts in the presence of a single transcription factor, Oct4, replacing Sox2, Klf4, and c-Myc (Li Y. et al., 2011). Based on these findings, a screen assay could be designed to identify alternative molecules that could replace Oct4, in combination with the small molecules, which could facilitate the establishment of wholly chemical iPSCs without any genetic modifications. More recently, a proof-of-principle study demonstrated that somatic reprogramming toward pluripotency could be manipulated using only small-molecule compounds, which revealed that the endogenous pluripotency program can be established by the modulation of molecular pathways nonspecific to pluripotency via small molecules rather than by exogenously provided “master genes” (Hou et al., 2013). The wholly chemical iPSCs enable the establishment of functionally desirable cell types in regenerative medicine by the employment of specific chemicals or drugs, instead of genetic manipulation, and difficult-to-manufacture biologics.

### Application of iPSCs in parkinson's disease

Incipiently, iPSCs are generated from mouse and human fibroblasts by enforced expression of the OSKM transcription factors, mediated by retrovirus or lentivirus, which might result in insertional mutagenesis, altered differentiation potential and virus-incurred immunoreaction and this would pose a risk for translational application in eventual PD treatment. Subsequently, in order to address these issues, previously employed retrovirus and lentivirus vectors have been substituted by non-integrating viral vectors and even non-viral vectors such as expressing plasmids, episomal vectors, minicircle vectors, polycistronic vectors/Cre-loxP system, piggyBac transposons, RNAs (mRNA and miRNA), protein and small molecules to generate mutagenesis- and integration-free iPSCs. However, in relation to the CRT for PD treatment, the application of iPSCs is regrettably lagging far behind the multifarious established iPSCs available, which is still by far confined to virus- and protein based iPSCs. Among the numerous iPSCs established by means of different vectors, virus-based iPSCs were primarily developed and most frequently applied in practical application of PD animal test. In 2008, Wernig et al. reported the reprogramming of mouse fibroblasts into iPSCs through retroviral transduction of the classical OSKM transcription factors, and the iPSCs derived DA neurons could synaptically and functionally integrate into a PD rat model (Wernig et al., 2008). In a following up study, patient specific iPSCs (PD-iPSCs)-derived TH expressing DA neurons could reduce motor asymmetry after transplantation into adult rodent brain, which was the maiden demonstration of human DA neurons derived from PD-iPSCs that could yield functional restoration in a preclinical model of PD (Hargus et al., 2010). Likewise, hiPSCs–derived NSCs/NPCs have also been proven to survive (Kikuchi et al., 2011) and morphologically and functionally integrate (Han et al., 2015) into the brain of transplanted PD primate (Kikuchi et al., 2011) and rats (Han et al., 2015) respectively and differentiate into neurons smoothly, including DA neurons *in vivo*. More recently, cynomolgus monkey iPSC-derived midbrain DA neurons have been certified to be capable of morphologically and functionally integrating into a non-human PD primate model after autologous unilateral engraftment, yielding a gradual onset of functional motor improvement contralateral to the side of DA neuron transplantation without a need for immunosuppression (Hallett et al., 2015). To overcome the potential safety issues associated with the employment of viruses, human piPSCs have been obtained without genetic manipulation and thus are emerging as a promising therapeutic source of CRT for PD treatment. Recently, two studies have demonstrated that human (Rhee et al., 2011) and murine (Kwon et al., 2014) piPSCs can efficiently generate functional DA neurons, respectively. Moreover, transplantation of the piPSCs derived DA neurons can significantly rescue motor deficits in PD rat models (Rhee et al., 2011; Kwon et al., 2014). Furthermore, these studies demonstrate that protein-based human iPSCs hold great promise to be a promising source of cells for clinical translation, these hiPSCs behave similar to hESCs without abnormal senescence/apoptosis, not showing any exogenous reprogramming gene expression, and DA neurons derived from human piPSCs significantly improving behavioral defects in a PD rodent model (see Table 3). In conclusion, as for the field of stem cell research and therapy relating to PD, more efforts and attention should be devoted to the multifariously established iPSCs apart from the existing virus- and protein-based iPSCs, which can tremendously boost the further development of CRT for PD treatment if implemented.

**Table 3:** **Summary of the application of iPSCs in PD animal models**.

**iPSCs**	**Grafted cells**	**PD models**	**Post-transplantation manifestations**	**References**
Virus-based iPSCs	DA neurons	Rat	Survival of grafted cells; expression of TH; relief of functional deficits	Wernig et al., 2008
PD-iPSCs	DA neurons	Rodent	Reduction of motor asymmetry; functional restoration	Hargus et al., 2010
Human iPSCs	NSCs	Primate	Smooth differentiation into DA neurons respectively; DA Synthesis and release	Kikuchi et al., 2011
	NPCs	Rat	Rescue functional deficits;	Han et al., 2015
Cynomolgus monkey iPSCs	DA neurons	Cynomolgus monkey	Survival of grafted neurons; extended neurite outgrowth; gradual onset of functional motor improvement	Hallett et al., 2015
Human piPSC	DA neurons	Rat	Survival of grafted DA neurons; rescue motor deficit significantly	Rhee et al., 2011
Murine piPSC	DA neurons	Rat	survival of grafted DA neurons; relief of functional deficits	Kwon et al., 2014

### Current status and future prospects

In general, establishment of ideal iPSCs converges on the applicable somatic cell sources, appropriate combination of reprogramming factors, efficient delivery methods and proper culture conditions. Depending on the purpose and application of iPSCs, choices concerning the somatic cell type, reprogramming factors, delivery method, and culturing conditions vary to substantial degrees (Brouwer et al., 2016), among which the reprogramming factors and delivery vectors have been elaborated in detail. On the basis of the protocols above, trans-differentiation from various somatic cells to iPSCs has been successfully established and the iPSCs derived DA neurons have been demonstrated to rescue motor deficits in PD animal models as well. In spite of the accomplishments above, there still exist limitations for generation of clinically feasible iPSCs.

#### Genetic and epigenetic alternations

The insertional or induced mutagenesis is an obvious restriction while employing integrating viral vector to establish iPSCs. Despite the non-integrating or non-viral delivery vectors can sharply reduce the risk of mutagenesis, several studies have identified de novo mutations during the reprogramming and culture process of iPSCs (Gore et al., 2011; Ji et al., 2012), thus resulting in genetic variation among generated iPSCs. Besides, the PD-iPSCs may also embody gene mutations such as point mutations, chromosomal structure variations, gene duplications, and deletions in the genes of SNCA, Parkin, LRRK2, GBA and so on (Mayshar et al., 2010; Gore et al., 2011; Laurent et al., 2011; Soldner et al., 2011; Reinhardt et al., 2013; Yu et al., 2015). Moreover, epigenetic modifications may also contribute to iPSC variation due to retained epigenetic memories of the initial cell type (Liang and Zhang, 2013).

#### Low reprogramming efficiency

The low reprogramming efficiency also remains a recurrent issue. Among the non-viral vector mediated iPSCs, RNA and protein based iPSCs appear to be promising alternatives, but its low reprogramming efficiencies compromise that possibility. On the contrary, the integrating viral vector mediated iPSCs demonstrate much higher yield in spite of potentially insertional mutagenesis. Hence, lentiviral vector, accompanied with favorable reprogramming efficiency, is still among the most successful reprogramming method (Schlaeger et al., 2015). Anyhow, future protocols should include comparisons between method reprogramming efficiencies, with the goal of optimizing protocols for maximum iPS cell yield.

Apart from the concerns above, the somatic cell type, delivery vectors and culture condition can exert considerable effect on the production of clinically feasible iPSCs. In addition, the induced-differentiation of iPSCs *in vitro* administration route and graft purity may count as well. Therefore, further optimization of establishment and induced-differentiation protocols remain a key gating item in translating iPSCs to the clinic for PD treatment in future study.

## Advantages and disadvantages of stem cell subtypes available in PD therapy

Various types of stem cells, including ESCs, NSCs, MSCs, and iPSCs, have been applied in basic and experimental studies relevant to PD, many of which have been translated to PD model transplantation, yielding miscellaneous and uneven outcomes. The preparation, induced differentiation, tentative application, current challenges and future prospects of the stem cells above have been elaborated, respectively. Then a comparison concerning advantages and disadvantages of the stem cell subtypes in PD therapy will be presented (see Table 4). ESCs, derived from the inner cell mass of a blastocyst, can totipotently differentiate into the three germ layers including nigral DA neurons (Lee et al., 2000). In addition, ESCs-derived DA neurons have demonstrated, after transplantation into PD animal models, extended neurite outgrowth, expression of specific synaptic markers and remmision of functional deficits. However, there still exist several serious concerns about the employment of these cells for PD treatment such as risk of tumor formation, ethical issues and immunological rejection, thus requiring more investigation to warrant further application in PD treatment. As for NSCs, one potential advantage over ESCs is that they are less prone to form tumors and incur immunological rejections after transplantation, apart from their restricted neural lineage differentiation. However, NSCs display several demerits such as limited differentiation capacity and sources, lingering ethical concerns, partial alleviation of Parkinsonian symptoms, sequela of GID (graft-induced dyskinesia) and emerging signs of senescence after repeated passagings (Ostenfeld et al., 2000). In contrast, several studies have shown that MSCs, absence of the demerits mentioned above, probably hold a much greater therapeutic potential for neurological diseases. Additionally, MSCs are more readily accessible, isolated and expanded more easily without immunorejection potential due to its lack of MHC-III (Morandi et al., 2008). Nevertheless, more investigations are imperative considering its biodistribution related toxicity, pale reprogramming efficiency and modest clinical improvement concluded from existing MSCs transplantation practices. In the case of iPSCs, the multifarious viral and non-viral delivery systems have enabled establishment of substantial integration-free iPSCs, thus immensely reducing the risk of tumor formation and insertional mutations. Moreover, iPSCs, especially PD-iPSCs, obtained directly from patients' somatic cells, are capable of dramatically reducing the risk of transmissible infections and minimizing relevant immunological rejections and ethical issues. However, the autologous transplantation of the patient specific iPSCs has the potential risk of being susceptible to the originally existed pathology of the patients. Besides, the contamination of a small portion of undifferentiated iPSCs or other unwanted cell lines has been proven to increase the risk of teratoma formation, decrease the reprogramming efficiency and compromise the ultimate function recovery when transplanted into PD animal models. In order to solve this issue, several stringent and effective sorting methods such as fluorescence-activated cell sorting (FACS; Wernig et al., 2008) or MACS (Rodrigues et al., 2014) have been developed to enable enrichment of differentiated neural cell lines and elimination of the undifferentiated iPSCs or other unwanted cell lines simultaneously. Thus, the integration of the FACS or MACS depletion step with the neural commitment protocol paves the way toward the establishment of a novel bioprocess for production of purified populations of hiPSC-derived neural cells for different applications, which can immensely boost the further development of high fidelity (Hi-Fi) iPSCs in CRT for PD treatment.

**Table 4:** **Advantages and disadvantages of stem cell subtypes in PD therapy**.

**Stem cell types**	**Source tissue**	**Advantages**	**Disadvantages**
Embryonic stem cells (ESCs)	Blastocyst	1. Totipotent differentiation 2. Preserve pluripotency after *in vitro* expansion 3. Forming all three germ layers 4. Generating midbrain DA neurons 5. Demonstrate to survive transplantation and rescue functional deficits	1. Tumorigenic hazards 2. Unaccessible source tissue/ethical concerns 3. Immunorejection 4. Biodistribution related toxicity
Neural stem cells (NSCs)	embryo/fetus/specific brain niches	1. Reduced risk of tumor formation and immunorejection in comparison with ESCs 2. Specific neural lineage differentiation into neurons, astrocytes, oligodendrocytes and DA neurons	1. Limited lineage differentiation *in vivo* 2. Modest functional recovery in PD model 3. Restricted source tissue/ethical concerns 4. Risk of GIDs 5.Biodistribution related toxicity
Mesenchymal Stem cells(MSCs)	Bone marrow, adipose tissue, umbilical cord, dermis, peripheral blood	1. Easily accessible source tissue 2. Rescue functional deficits in mice 3. Rare adverse effects in humans following transplantation 4. Considerable pluripotency	1. Modest functional recovery in humans 2. Biodistribution related toxicity
Induced pluripotent stem cells(iPSCs)	Somatic cells/differentiated cells	1. Remarkable pluripotent differentiation 2. Histocompatibility, suited for autologous transplantation 3. Survive transplantation and rescue functional deficits 4. Easily accessible source tissue 5. Minimal immunorejection and ethical issues	1. Tumorigenic hazards 2. Susceptibility to donor's genetic mutation associated with autologous transplantation 3. Low iPSCs yield 4. Biodistribution related toxicity

## Interplay between CRT practice and systemic immunity

**Figure 5:**
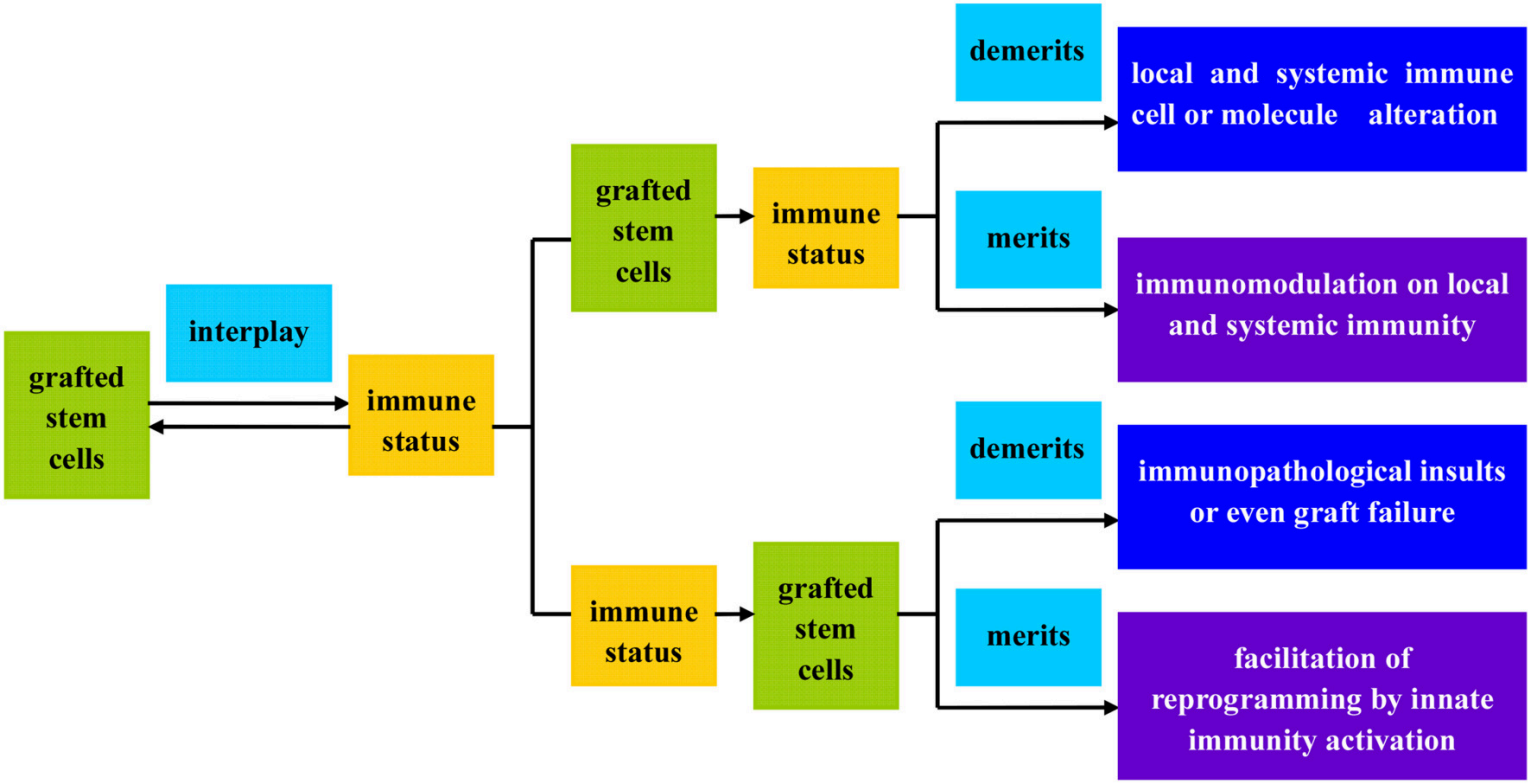
**Flow illustration of the interplay between grafted stem cells and recipient immunity**. The grafted stem cells and recipient immunity can form a functional entity upon transplantation, exerting reciprocal impacts, merits, and demerits, on each other.

As stated above, the stem cells available hold great promise for CRT of PD treatment in spite of mixed blessings, but the concomitant consequence on immunity is another grave concern. The allogeneic grafts usually evoke intense immunological rejection inevitably, taking a heavy toll on the grafts and recipients or even resulting in transplantation failure if not intervened properly. A good case in point is the initial transplantation of hfVM and its derivatives (eg. ESCs) into patients suffering from neurodegenerative diseases (Tomaskovic-Crook and Crook, 2011). But the unpleasant situation gradually improves with the emergence of cell engineering and newly developed immunosuppressants, especially the derivation and application of autologous MSCs and PD-iPSCs which, by and large, solve the conundrum of graft induced immunorejection. In fact, the grafted stem cells and recipients unite as a heterogenous entity upon transplantation, exerting reciprocal impacts, merits and demerits, on each other (see Figure 5).

Neuroglial cells such as astrocytes and microglia have been increasingly recognized as possessing crucially physiological and pathological functions within brain, especially in relation to neurodegenerative diseases such as PD, Alzheimer's Disease and Amyotrophic Lateral Sclerosis (Minghetti, 2005; Gao and Hong, 2008; Glass et al., 2010), which have been, therefore, hypothesized as neuroinflammatory disorders. Nevertheless, neuroinflammation have been accepted as a double-edged sword executing both beneficial and detrimental effects on neurons and glial cells (Hanisch and Kettenmann, 2007; Tang and Le, 2014). The grafted cells, especially allogeneic origins, have been shown to secret a plethora of cytokines that can directly modulate phenotype switch of immune cells both in local tissue and in the systemic circulation (Kassis et al., 2008; Coulson-Thomas et al., 2016), thus resulting immunopathological damages to the grafts and recipients as well (see Figure 5). Indeed, the immunity activation incurred by allogeneic grafts is not always pernicious. For example, it has recently demonstrated that innate immunity activation can facilitate the expression of epigenetic modifiers and eventually enhance nuclear reprogramming efficiency (Lee et al., 2012). Stimulation of toll-like receptor 3 (TLR3) signaling pathway appear to enable recipient cell to adopt an open chromatin configuration which can increase cell plasticity to external pathogens, thus contributing to efficient induction of pluripotency by viral or mmRNA approaches (Lee et al., 2012). Hence, it can be speculated that this innate immunity triggered global epigenetic modification may enhance transdifferentiation, disdifferentiation or even malignant transformation, a process has been termed “transflammation” (Lee et al., 2012).

**Figure 6:**
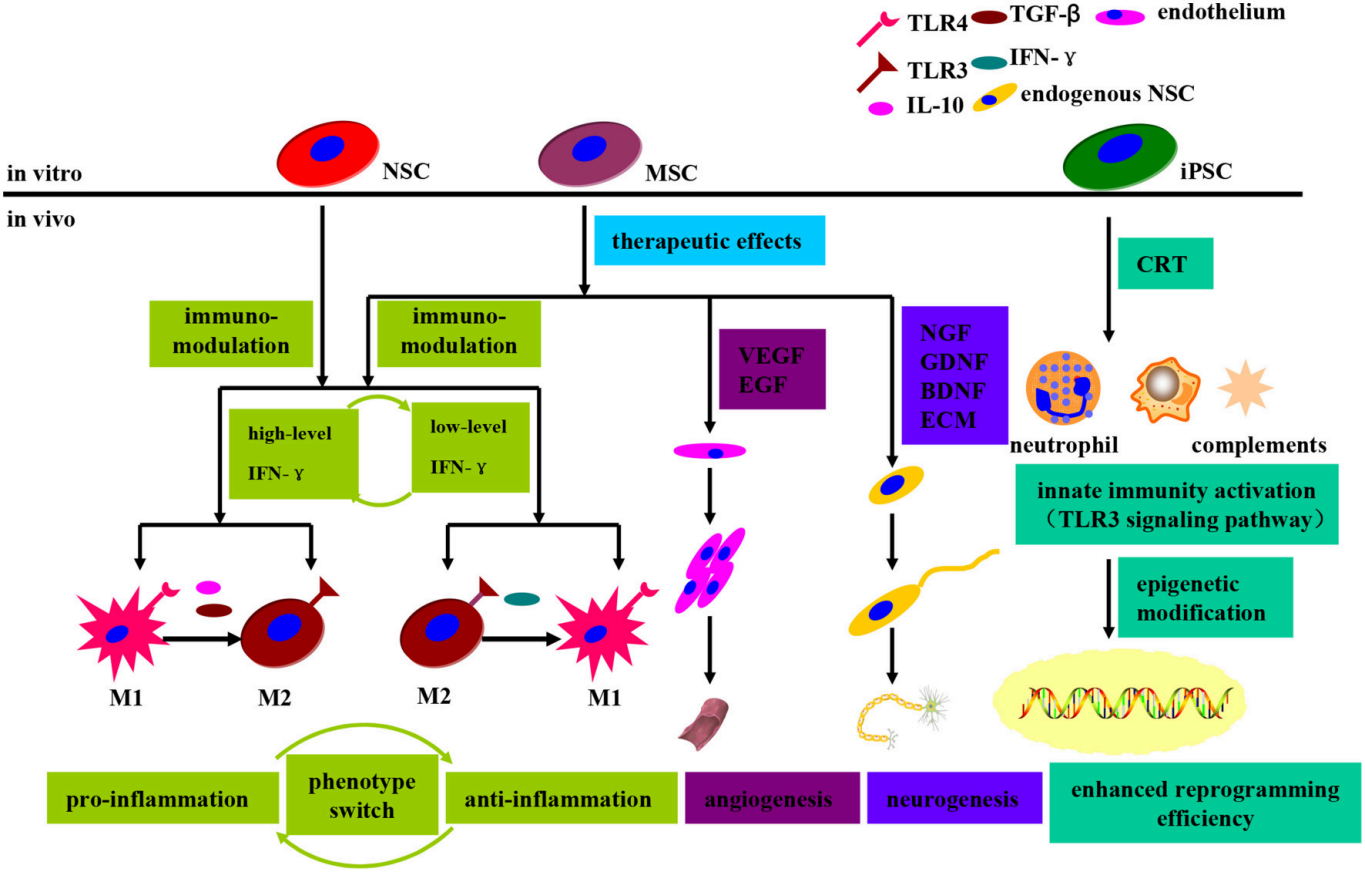
**Schematic diagram demonstrating the immunomodulatory and therapeutic effects that grafted stem cells exerted on the recipient upon transplantation**. The immunomodulatory effects, regulated by MSCs and NSCs, are dominated by the delicate balance of IFN-γ and executed by the microglial phenotype switch between M1 and M2. An anti-inflammatory microenvironment can promote microglial TLR4 expression and IFN-γ secretion in MSCs, thus switching to a pro-inflammatory state. Similarly, a pro-inflammatory microenvironment can switch to an opposite one via microglial TLR3 expression upregulation and anti-inflammatory cytokines secretion. Moreover, MSCs can promote angiogenesis and neurogenesis through secretion of VEGF, EGF, and neurotrophic factors. In the context of iPSCs transplantation, the innate immunity activation (TLR3 signaling pathway activation) can enhance nuclear reprogramming efficiency via facilitation of epigenetic modifiers.

Apart from that, the grafted cells, especially MSCs and NSCs, have been shown to be able to exert a regulatory effect on adaptive and innate immune cells. The earliest report describing immunomodulatory property was by Le Blanc in relation to MSCs, who found that transplantation of allogeneic MSCs could suppress immune cells and prevent the graft-vs.-host attack (Le Blanc, 2003). To date, MSCs have been proven to exert immunomodulation effects primarily in a cytokine dependent manner through secretion of transforming growth factor-beta (TGF-β; James et al., 2005), interferon gamma (IFN-γ; Aggarwal and Pittenger, 2005), tumor necrosis factor-alpha (TNF-α; James et al., 2005), IL-10 (Niu et al., 2014) and et al. Furthermore, the microglial M1 and M2 phenotype switch have been shown to be implicated in the immunomodulation process as well (Coulson-Thomas et al., 2016; see Figure 6). An anti-inflammatory environment marked by low level of IFN-γcan promote microglial TLR4 expression and IFN-γsecretion in MSCs, thus creating a pro-inflammatory microenvironment. To the contrary, a pro-inflammatory microenvironment (high level of IFN-γ) can switch microglial M1 plorization to M2 type via secretion of anti-inflammatory cytokines such as IL-10, TGF-β and et al., thus fostering an anti-inflammatory microenvironment and inhibiting neutrophil, T-cell and microglia recruitment (Coulson-Thomas et al., 2016; see Figure 6). Therefore, the MSCs immunomodulation effects are dominated by the delicate balance of IFN-γ and executed by microglial phenotype switch. Besides, NSCs transplantation into Alzheimer's Disease model mice has demonstrated significant cognitive deficits improvement via attenuation of glial activation and pro-inflammatory TLR4 signaling pathway, indicating that neuroinflammation contributes to cognitive impairment and neuroinflammation targeted therapy can be developed to prevent or delay Alzheimer's Disease progression (Zhang et al., 2015). Moreover, intracerebral transplantation of primed hNSCs has efficiently induced a transition of microglia toward anti-inflammatory M2 phenotype, which presumably contributes to stem cell mediated neuroprotection after severe brain injury in mice (Gao et al., 2016). As shown above, the immunomodulatory effects demonstrated by MSCs and NSCs can be utilized for the treatment of autoimmune and degenerative diseases.

Among the numerous therapeutics available, the stem cell replacement therapy (CRT) holds a gleam of promise for PD restorative treatment. What is frustrated is that the concomitant graft induced immunopathological injuries or even immunorejection have cast a shadow on this promising therapeutic regimen. Nevertheless, the immunomodulatory effects revealed by the stem cells, especially MSCs and NSCs, are of substantial potential to become bran-new therapeutic targets for PD treatment, more than just providing differentiated DA neurons in CRT practice. Therefore, to circumvent the allogeneic graft induced immunal damages, there are three countermeasures that we can adopt to deal with it: (i) guaranteeing favorable and eligible transplantation zygosity; (ii) developing more effective and accessible suppressants; (iii) making full exploitation of the immunomodulatory stem cells, especially MSCs and NSCs. To sum up, future efforts should be concentrated to foster the strength and circumvent the weakness so as to mold stem CRT into a secure, reliable and generalized therapeutic strategy for PD treatment.

## Conclusions

Currently, treatment and medications available for PD conformably intend to rescue motor deficits by providing dopamine substitutes or DA receptor agonists and in advanced PD patients, DA synergistic agents, control-released L-dopa and even DBS. In spite of significant rescue and alleviation of functional deficits, the pharmaco-therapeutic effects are gradually undermined, implicated by various types of motor response oscillations such as on-off, wearing off phenomena, as well as LID. In this stretched context, stem cell based CRT has emerged as a restorative therapy for PD. The existing CRT practices have demonstrated survival, regeneration, replacement and re-innervation of DA neurons in vulnerable lesions, thus holding enormous promise to be a restorative therapy in PD. Besides, the stem cell research and CRT has led to several novel insights in relation to PD pathogenesis. Of most importance, the evidence that Lewy bodies can propagate from host cells to graft cells has prompted research to investigate the hypothesis that PD is a prion like disease. In addition, neuroinflammation is a common feature shared by various neurodegenerative diseases such as PD, Alzheimer's Disease and Amyotrophic Lateral Sclerosis, so the immunomodulatory features demonstrated by the stem cells can be used to corroborate the neuroinflammation hypothesis of PD pathogenesis. Hence, stem cell research will boost not only CRT in PD, but also etiological elucidation as well.

Despite the fact that CRT is a promising avenue for the treatment of PD and other neurodegenerative disorders, there still exist numerous hurdles, such as tumorigenesis, immune response, low stem cell yield and bio-distribution related toxicity, to be straightened out so as to enable development of novel approaches that could be translated into effective and universally accepted clinical application. Apart from that, the disease stages and severity may also have an impact on the effect of CRT. In conclusion, the CRT practice is still in its infancy, so it is imperative for us to establish more competent stem cell lines in future studies so as to propel the stem cell research and therapy, in relation to PD, from bench to bed.

## Author contributions

YS, JH are responsible for the the conception, pencraft, and modification of the manuscript; LL, XX, CH, GZ, HJ, JL equally contribute to the collection and sorting of the data and documents; ZL, NX manipulate the polishing of the language and writing style; TW manages the integral envisage and top-layer design of the manuscript.

### Conflict of interest statement

The authors declare that the research was conducted in the absence of any commercial or financial relationships that could be construed as a potential conflict of interest.

## Abbreviations

BDNF: brain derived neurotrophic factor
L-DOPA, L-3: 4-dihydroxy-phenylalanine
bFGF: basic fibroblast growth factor
LIF: leukemia inhibitory factor
BMSCs: bone mesenchymal stem cells
MEFs: mouse embryonic fibroblasts
cDNAs: complementary DNAs
MSCs: mesenchymal stem cells
CNS: central nervous system
NGF: nerve growth factor
CPP: cell penetrating peptide
NPCs: neural progenitor cells
CRT: cell replacement therapy
NSCs: neural stem cells
DA: dopaminergic
ONSL, Oct4, Nanog, Sox2: Lin28
DBS: deep brain stimulation
OSKM, Oct3/4, Sox2, Klf4: c-Myc
Ebs: embryoid bodies
PCR: polymerase chain reaction
ECM: extra cellular matrix
PD-iPSCs: PD patient specific iPSCs
EGF: epidermal growth factor
piPSCs: protein-based iPSCs
ESCs: embryonic stem cells
PD: Parkinson's Disease
FGF: fibroblast growth factor
SeV: Sendai Virus
GDNF: glial cell derived neurotrophic factor
SDIA: stromal cell derived inducing activity
GID: graft induced dyskinesia
SNpc: substantia nigra pars compacta
GMP: good manufacturing practice
SVZ: subventricular zone
hESCs: human embryonic stem cells
TFs: transcription factors
hfVM: human fetal ventral midbrain
TH: tyrosine hydroxylase
hiPSCs: human induced pluripotent stem cells
TLR3/4: toll-like receptor3/4
IL: interleukin
VEGF: vascular endothelial growth factor
iNSCs: induced neural stem cells
VPA: valproic acid
iPSCs: induced pluripotent stem cells
VM: ventral midbrain
LID: L-DOPA induced dyskinesia.
